# Evaluation of Digital Tomosynthesis for Detecting Articular Cartilage Lesions in Equine Distal Interphalangeal and Metacarpophalangeal Joints: Comparison with Computed Tomography Arthrography

**DOI:** 10.3390/ani16213398

**Published:** 2026-10-28

**Authors:** Alisa Hahn, Miguel Valdés Vázquez, Gabriel Manso-Díaz, Dagmar Berner, Joana Maia Ramos, Marcus G. Doherr, Christoph J. Lischer

**Affiliations:** 1 Equine Clinic, Veterinary Hospital, School of Veterinary Medicine, Freie Universität Berlin, Oertzenweg 19b, 14163 Berlin, Germany; christoph.lischer@fu-berlin.de; 2 Hospital de Referencia La Equina, Apdo. 110, Camino de Martagina km 1, 29692 Manilva, Spain; miguel@laequina.com (M.V.V.); joana.126@hotmail.com (J.M.R.); 3 Department of Animal Medicine and Surgery, Faculty of Veterinary Medicine, Complutense University of Madrid, Avenida Puerta de Hierro s/n, 28040 Madrid, Spain; gmanso@ucm.es; 4 Equine Referral Hospital, Department of Clinical Science and Services, Royal Veterinary College, Hawkshead Lane, North Mymms, Hatfield AL9 7TA, Hertfordshire, UK; 5 Institute of Veterinary Epidemiology and Biostatistics, School of Veterinary Medicine, Freie Universität Berlin, Königsweg 67, 14163 Berlin, Germany; marcus.doherr@fu-berlin.de

**Keywords:** osteoarthritis, diagnostic performance, contrast-enhanced imaging, simulated weight-bearing, fetlock joint, coffin joint, interobserver agreement, ex vivo study

## Abstract

Conventional radiography has limited sensitivity for detecting equine cartilage lesions. This prospective ex vivo study compared digital tomosynthesis (DT), computed tomography (CT), digital tomosynthesis arthrography (DTA), and computed tomography arthrography (CTA) for detecting artificial and naturally occurring partial- and full-thickness cartilage lesions in distal interphalangeal and metacarpophalangeal joints. Sixteen distal forelimbs from nine horses were examined under simulated weight-bearing (SWB) and non-weight-bearing (NWB) conditions. Three blinded observers evaluated 24 regions per limb, using gross examination as the reference standard. Sensitivity, specificity, and agreement were calculated, and mixed-effects logistic regression was performed. Gross examination identified lesions in 141 of 384 regions. Mean sensitivities (95% confidence intervals), reported for SWB and NWB, respectively, were highest for CTA [60.8% (56.7–64.9); 57.0% (53.4–60.5)], followed by DTA [14.7% (10.6–18.7); 29.1% (24.5–33.9)], CT [13.0% (9.3–16.7); 15.4% (12.0–18.8)], and DT [0.5% (0.0–1.0); 1.7% (0.9–2.4)]. Mean specificities ranged from 96.8% (95.3–98.4) to 99.7% (99.3–100.0). For agreement with gross examination, CTA had the highest pooled Cohen’s κ. Contrast medium administration was associated with higher odds of true-positive and lower odds of true-negative classification. Weight-bearing did not consistently improve lesion detection. Clinical performance requires evaluation in living horses.

## Simple Summary

Osteoarthritis can damage the smooth cartilage covering the joints of horses, leading to pain and lameness. Cartilage damage can be difficult to detect because cartilage is not directly visible on standard X-rays. This study compared digital tomosynthesis and computed tomography for detecting cartilage lesions in the coffin and fetlock joints. Both methods were assessed before and after injection of a contrast medium to outline the cartilage surface. Sixteen lower forelimbs collected from nine horses were examined after death, both with and without simulated loading. Three veterinarians assessed the images, and their findings were compared with direct inspection of the cartilage. Computed tomography after contrast medium injection detected the greatest proportion of cartilage lesions. More lesions were detected after contrast medium injection, but regions without lesions were also more often incorrectly classified as damaged. Digital tomosynthesis without contrast medium detected very few lesions. Simulated loading showed no consistent benefit. For digital tomosynthesis, differences in joint position and imaging direction prevented the effect of loading from being assessed separately. These findings apply to the experimental conditions used. Further studies in living horses and comparisons with conventional radiography or magnetic resonance imaging are needed.

## 1. Introduction

Articular cartilage lesions are a key feature of equine osteoarthritis (OA), one of the leading causes of lameness, particularly in athletic horses [1,2,3]. The metacarpophalangeal (MCP) [2,4,5] and distal interphalangeal (DIP) [6,7,8] joints are among the joints most frequently affected by OA [1,9,10,11,12]. Accurate detection of articular cartilage lesions is crucial for diagnosis and treatment planning [13,14,15]; however, early assessment remains challenging because conventional radiography cannot directly visualise articular cartilage [16].

Cross-sectional imaging techniques have improved the evaluation of equine joints [17]. Magnetic resonance imaging (MRI) provides excellent soft-tissue contrast, whereas computed tomography (CT) offers superior assessment of osseous structures [17,18]. Following intra-articular contrast medium administration, computed tomography arthrography (CTA) has emerged as one of the most accurate imaging techniques for detecting focal articular cartilage defects [19,20,21]. In standing horses, CT examinations of the distal limb, including the foot and MCP joint, have also been performed using a multi-slice helical scanner [22].

Digital tomosynthesis (DT) is an emerging imaging modality that has gained increasing attention in human musculoskeletal imaging for disorders of the knee [23], wrist [24], and hand [25,26]. The use of DT as an intermediate modality between conventional radiography and CT has been investigated [27,28]. By acquiring a series of low-dose radiographic projections over a limited angular range, DT reconstructs multiple sectional images while generally exposing the patient to less radiation than CT [28]. Unlike conventional radiography, DT reduces the problem of superimposed anatomical structures, thereby improving the visualisation of osseous abnormalities [27,28]. In human medicine, DT has been shown to improve the detection of musculoskeletal lesions, including bone erosion associated with rheumatoid arthritis [25,26], and its diagnostic performance has approached that of MRI in some applications [25].

In a cadaveric study of equine MCP joints, Stewart et al. [29] identified a subchondral bone lesion of the third metacarpal bone (MC3) with DT that was not visible on radiographs. Compared with DT, MRI provided better visualisation of articular cartilage. The authors suggested that DT may serve as a complementary imaging modality for detecting subtle subchondral bone lesions [29].

The ability of DT to generate sectional images rapidly using conventional X-ray technology, combined with its portability, low radiation dose, and potential for standing examinations under minimal sedation, makes it an attractive imaging modality for equine practice [29]. However, the diagnostic performance of DT for detecting articular cartilage lesions in horses has not yet been established.

Therefore, the objective of this study was to compare the diagnostic performance of DT, CT, digital tomosynthesis arthrography (DTA), and CTA for detecting artificial and naturally occurring articular cartilage lesions in the equine DIP and MCP joints, using gross pathological assessment as the reference standard. In addition, the effects of intra-articular contrast medium administration and simulated weight-bearing (SWB) on lesion detection were evaluated.

We hypothesised that CTA would have the highest sensitivity for the detection of articular cartilage lesions, followed by DTA, CT, and DT. Furthermore, we hypothesised that intra-articular contrast medium administration and SWB would improve lesion detection.

## 2. Materials and Methods

Sixteen distal forelimbs were collected from nine adult horses by disarticulation at the middle carpal joint. The specimens comprised eight right and eight left forelimbs. Seven horses contributed both forelimbs, and two contributed one forelimb each. Each limb was assessed separately, including paired limbs from the same horse. Median age was 13 years (first-third quartiles [Q1–Q3], 12–17 years; range, 3–20 years), and median body weight was 500 kg (Q1–Q3, 500–570 kg; range, 480–650 kg). The group comprised four mares, four geldings and one stallion. The study population comprised Hanoverian (*n* = 2), German Sport Horse (*n* = 2), Thoroughbred (*n* = 2), Holsteiner (*n* = 1), Arabian (*n* = 1), and Pura Raza Española (*n* = 1) horses. All horses were euthanised for reasons unrelated to musculoskeletal pathology: severe gastrointestinal disease (*n* = 7), haemolytic anaemia (*n* = 1), and severe neurological deficits (*n* = 1). No horse was euthanised for the purpose of this study.

The limbs were collected at the Equine Hospital, Freie Universität Berlin, within 12 h of euthanasia. The surrounding soft tissues were preserved during disarticulation. Each specimen was labelled, wrapped in plastic film and frozen at temperatures below −20 °C. Before further processing, the specimens were thawed at room temperature for approximately 12 h under the same conditions. They were then cleaned and clipped, and any horseshoes were removed.

Details of the individual horses and limb specimens are presented in the Supplementary Table S1. Ethical approval was granted by the local research ethics committee (StN 046/23, 16 January 2024).

### 2.1. Experimental Design

Sixteen distal forelimbs from nine horses were sequentially imaged using four imaging protocols (DT, CT, CTA, and DTA) under both SWB and non-weight-bearing (NWB) conditions. Artificial cartilage lesions were created according to a predefined randomisation protocol, while naturally occurring lesions were documented during gross examination. Gross pathology served as the reference standard.

No formal a priori sample size calculation was performed. This was an exploratory ex vivo study, and no directly applicable preliminary estimates of DTA diagnostic performance were available to support a reliable calculation incorporating the repeated assessments within horses, limbs and anatomical regions.

### 2.2. Creation of Cartilage Lesions

Artificial cartilage lesions were created in both the MCP and the DIP joints. An investigator not otherwise involved in the study prepared a predefined randomisation scheme specifying the number, anatomical location and intended depth of cartilage lesions for each joint, as well as the allocation of the sham joint. Between one and four partial- or full-thickness cartilage lesions were planned for each joint assigned to lesion creation. A sham surgical approach without intentional creation of cartilage lesions was performed in one MCP joint. Regions within this joint were classified according to their gross pathological findings, including any naturally occurring cartilage lesions. Lesions were created through one dorsal arthrotomy per joint via the digital extensor tendon.

**Figure 1:**
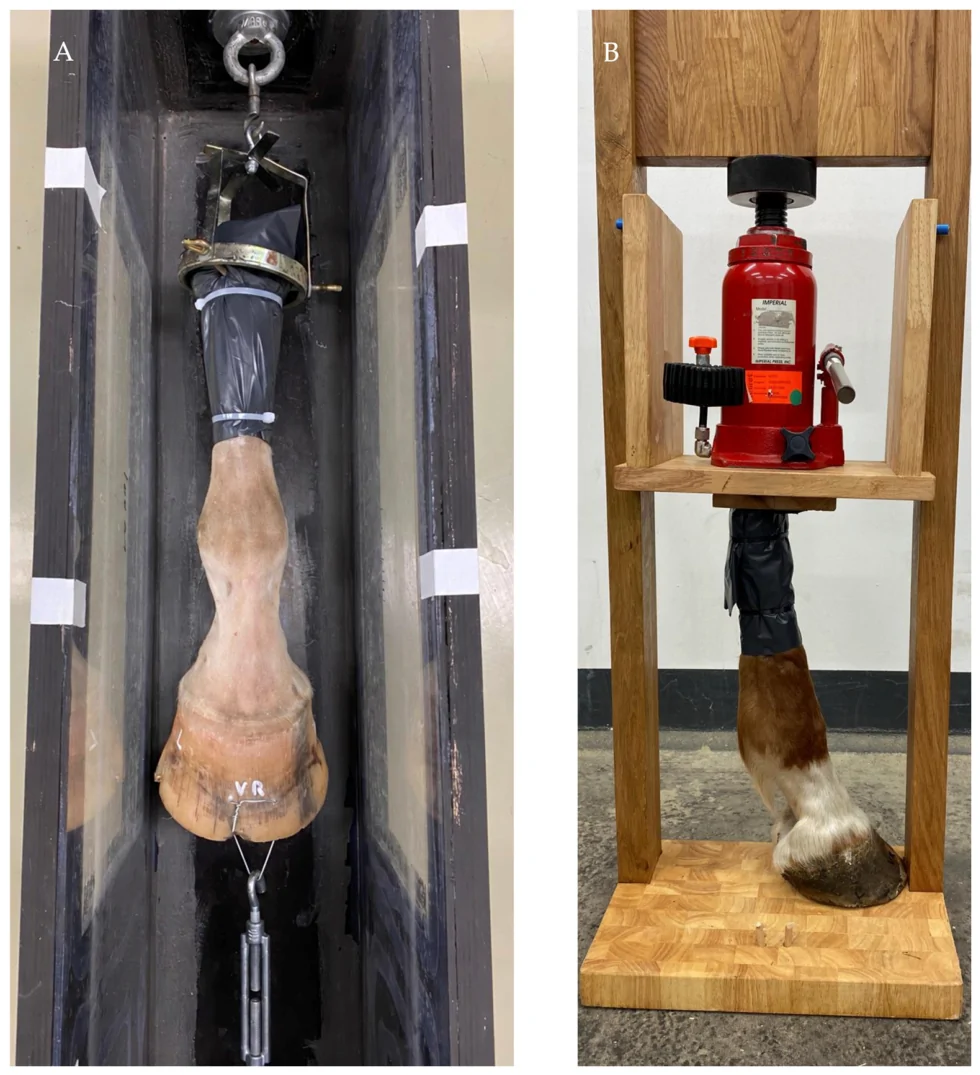
Experimental setups for cartilage lesion creation and simulated weight-bearing (SWB) conditions. (**A**) Custom-designed basin and traction device used for cartilage lesion creation. The proximal end of the limb was secured in a fixation device, and longitudinal traction was applied using a tensioning mechanism connected to a wire loop passing through two holes in the distal hoof wall to maximise joint distraction. (**B**) Custom-made loading frame with a hydraulic jack used to apply axial load during imaging under SWB conditions.

To maximise joint distraction and facilitate access to articular surfaces, the cadaver limb was placed under longitudinal tension. For this purpose, two holes were drilled in the distal hoof wall, and a loop made from 2-mm-diameter wire was inserted and twisted. To minimise the introduction of intra-articular air, the limb was connected to a custom-designed underwater basin. The proximal end of the limb was then secured in a custom-made fixation device, and longitudinal traction was applied until maximum joint distraction was achieved (Figure 1).

Access to the MCP and DIP joints was established using 20-gauge needles inserted through the extensor tendon. Correct intra-articular needle placement was confirmed by digital radiography. A portal was then created with a No. 11 scalpel blade to allow insertion of a Volkmann bone curette. Two digital radiographs were obtained per joint: one to confirm intra-articular needle placement and one to confirm intra-articular curette placement. The needle and curette remained in place without manual support during exposure. Exposures were initiated from a shielded position outside the room [30]. Manipulation and lesion creation were performed with the beam off. The curette was then directed medially, laterally or axially towards the planned lesion location. For partial-thickness lesions, the aim was to keep the defect confined to the cartilage without reaching the subchondral bone. For full-thickness lesions, the aim was to remove cartilage down to the subchondral bone. Lesions were created without direct visualisation of the articular surfaces.

After lesion creation, the limb was removed from the fixation device. The joint capsule was sealed with hot-melt adhesive, and the skin was closed with 2–0 polyglactin 910 using a V-34, 36 mm, 1/2 circle needle (Ethicon, Somerville, NJ, USA).

Actual lesion depth was determined after imaging by gross examination, as described in Section 2.4.

### 2.3. Imaging Protocol

Following lesion creation, the limbs were maintained at room temperature throughout all imaging procedures. Each limb underwent native DT, native CT, CTA and DTA in this fixed order, with SWB acquisitions preceding NWB acquisitions for each protocol. The approximate total examination times per limb, including both loading conditions, were 30 min for native DT, 10 min for native CT, 10 min for CTA and 30 min for DTA.

For SWB acquisitions, the limbs were mounted in a custom-built wooden loading frame incorporating a hydraulic jack, as described by Hahn et al. [31] (Figure 1, panel B). Static axial loading was applied to the proximal end of each limb. The target load was based on a reference load of 1000 N per limb for a 450-kg horse [32] and was scaled to the mean body weight of the horses in the present study (528 kg), yielding a calculated target force of 1173 N. Axial force was calculated from hydraulic pressure using F = pA, with a piston cross-sectional area of 91.6 cm^2^, calculated from a piston diameter of 10.8 cm. The pressure was monitored using the integrated gauge and restored to the same target reading after transfer or repositioning. The axial force was not independently verified using a separate force-measuring device. The limbs were positioned vertically for DT and in lateral recumbency on the CT table for CT.

The DT images were acquired with the EqueTom™ system (Orimtech Ltd., Buffalo Grove, IL, USA), equipped with a Poskom PXM-20BT X-ray unit (POSKOM Co., Ltd., Goyang-si, Republic of Korea) and an Iona2-RF-2430 dynamic detector (Teleoptic PRC Ltd., Kyiv, Ukraine). The detector had a field of view of 244 × 304 mm and 16-bit digitisation. Each acquisition comprised 120 radiographic projections obtained over 3.2 s at 81 kVp and 50 mAs. The angular sweep used for the study acquisitions was not available from the acquisition records. Reconstruction with Orimtech’s Ace-Clubs software produced an initial stack of 340 slices with a slice thickness of 0.6 mm, a matrix of 1520 × 1280 pixels and an in-plane pixel spacing of 0.18 × 0.18 mm.

Under SWB conditions, lateromedial (LM) and dorsopalmar (DPa) DT acquisitions were obtained for both the MCP and DIP joints. A radiodense marker was placed on the lateral aspect of the limb to facilitate image orientation. Under NWB conditions, LM projections of both joints were acquired with the joints slightly flexed. In addition, slightly flexed DPa projections were obtained for the MCP joint, whereas a dorsoproximal–palmarodistal oblique projection was obtained for the DIP joint. For the flexed acquisitions, the hoof was positioned on a Hickman block [33]. Native DT examinations were performed before intra-articular contrast medium administration, resulting in a total of eight native DT acquisitions per limb.

For CT, imaging extended from the mid-metacarpus to the hoof and was performed using an Aquilion/LB fan-beam multidetector scanner (Toshiba Medical Systems Corporation, Otawara, Japan). Helical acquisition settings were 120 kVp, 250 mA, a rotation time of 0.75 s, a pitch of 0.688 and a collimation of 16 × 1.0 mm. Reconstruction was performed with Adaptive Iterative Dose Reduction 3D (Canon Medical Systems Corporation, Otawara, Japan) at the Mild setting using bone and soft-tissue kernels. The reconstructed images had a slice thickness of 1.0 mm, a reconstruction interval of 0.8 mm, a field of view of 240 × 240 mm, a matrix of 512 × 512 pixels and an in-plane pixel spacing of 0.468 × 0.468 mm. The limbs were positioned in lateral recumbency on the CT table, with a plastic needle cap marking the lateral aspect of the hoof. Native CT examinations were acquired first under SWB and then under NWB conditions, providing two native scans per limb. Within each modality, the acquisition and reconstruction parameters were identical for SWB and NWB examinations and before and after intra-articular contrast medium administration.

Subsequently, CT arthrography was performed following intra-articular injection of a mixture of contrast medium (Solutrast^®^, iopamidol; 200 mg I/mL, Bracco Imaging Deutschland GmbH, Konstanz, Germany) and 0.9% NaCl at a ratio of 3:1 (contrast medium to saline), yielding a final iodine concentration of 150 mg I/mL. Each MCP joint received 25 mL of the mixture, and each DIP joint received 15 mL. To promote homogeneous distribution of contrast medium [34], each distal limb was moved through 20 cycles of extension and flexion before CT imaging was repeated. The complete CT protocol therefore consisted of four examinations per limb: SWB native, NWB native, SWB contrast-enhanced, and NWB contrast-enhanced.

Following completion of CTA, eight DTA acquisitions were obtained for each limb under SWB and NWB conditions, without additional contrast medium administration or repeated flexion–extension cycles.

**Figure 2:**
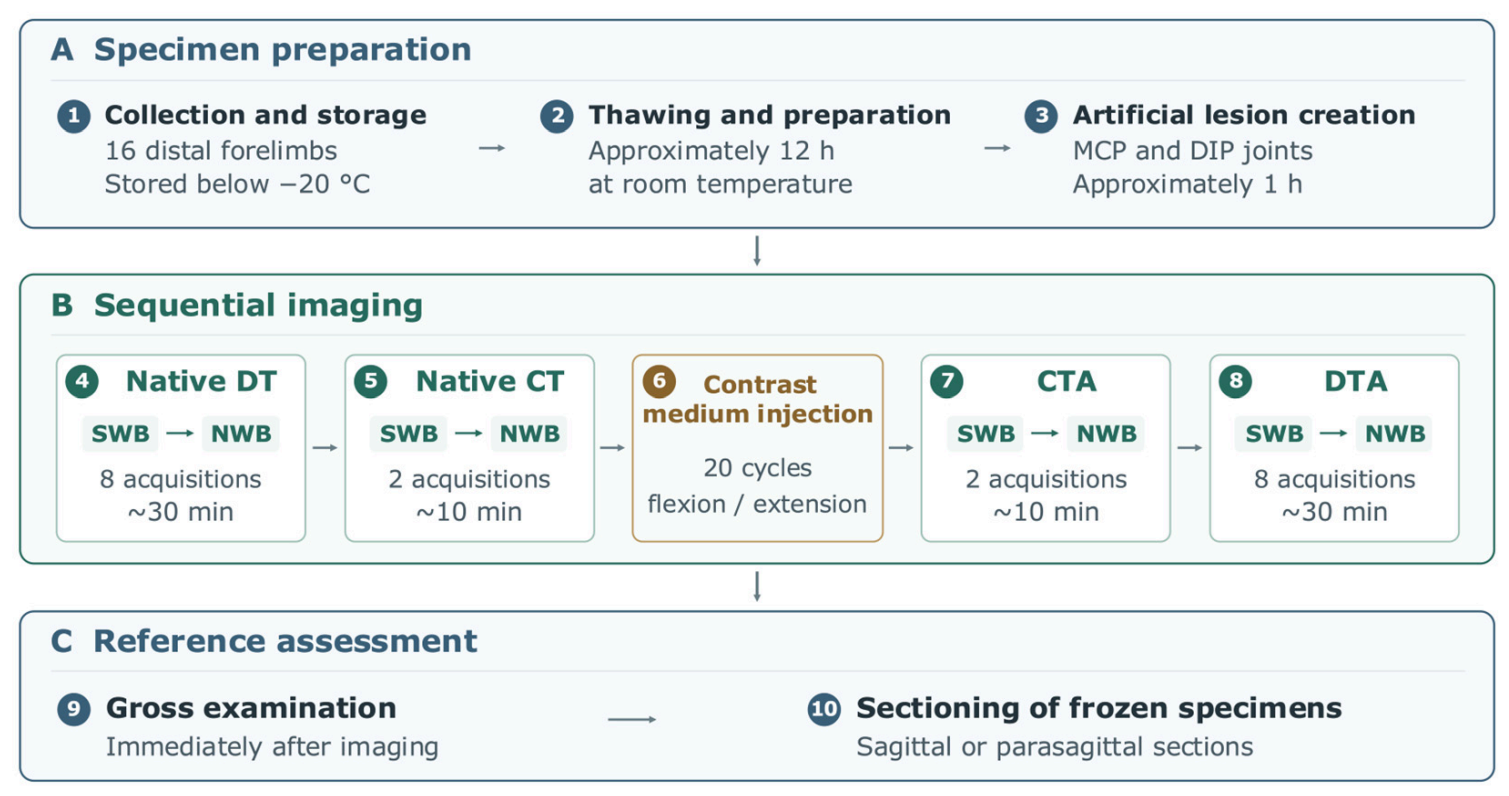
Experimental workflow for each specimen. (**A**) Specimen preparation, including collection, storage, thawing and artificial lesion creation. (**B**) Sequential imaging under simulated weight-bearing (SWB) and non-weight-bearing (NWB) conditions. (**C**) Gross examination and sectioning of frozen specimens. MCP, metacarpophalangeal; DIP, distal interphalangeal; DT, digital tomosynthesis; CT, computed tomography; DTA, digital tomosynthesis arthrography; CTA, computed tomography arthrography.

The same joint positioning and projections were used as for native DT. The loading apparatus was readjusted between examinations. The complete imaging workflow is summarised in Figure 2.

### 2.4. Gross Analysis

**Figure 3:**
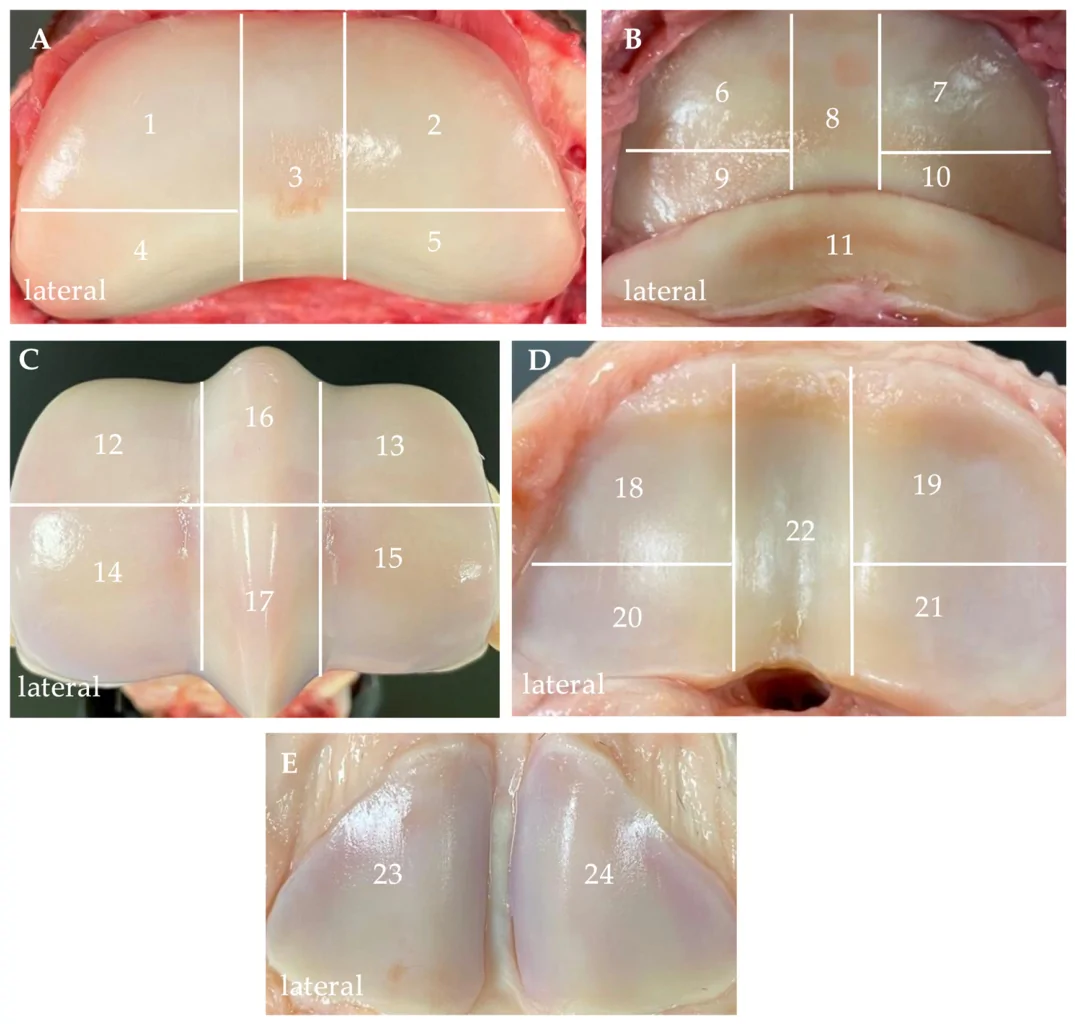
Anatomical regions used for the gross pathological assessment of articular cartilage lesions. The articular surfaces of the MCP and DIP joints were divided into 24 predefined anatomical regions. The lateral aspect of each specimen is indicated. (**A**) The articular surface of the middle phalanx (MP) was divided into five regions: dorsolateral (1), dorsomedial (2), axial (3), palmarolateral (4), and palmaromedial (5). (**B**) The articular surface of the distal phalanx (DP) was divided into five regions: dorsolateral (6), dorsomedial (7), axial (8), palmarolateral (9), and palmaromedial (10). (**B**) The navicular bone was assessed as a separate region (11). (**C**) The articular surface of the third metacarpal bone (MC3) was divided into six regions: dorsolateral (12), dorsomedial (13), palmarolateral (14), palmaromedial (15), dorsal sagittal ridge (16), and palmar sagittal ridge (17). (**D**) The articular surface of the proximal phalanx (PP) was divided into five regions: dorsolateral (18), dorsomedial (19), palmarolateral (20), palmaromedial (21), and sagittal groove (22). (**E**) The proximal sesamoid bones were assessed as two separate regions: lateral (23) and medial (24). MCP, metacarpophalangeal; DIP, distal interphalangeal.

Gross examination was performed immediately after imaging. The limbs were carefully dissected by transecting the collateral ligaments and disarticulating the joints. All articular surfaces were examined macroscopically across 24 predefined anatomical regions (Figure 3). All 384 anatomical regions were examined macroscopically and were assessable on gross examination. Each articular surface was photographed, and both artificially created lesions and naturally occurring cartilage lesions were identified and documented. Following the initial gross examination, the specimens were frozen to facilitate cutting. With assistance from a specialist from the Institute of Pathology at Freie Universität Berlin, serial sagittal or parasagittal sections were obtained from the frozen specimens to determine lesion depth. Each anatomical region was classified as having no cartilage lesion, a partial-thickness lesion, or a full-thickness lesion. Full-thickness lesions extended to the subchondral bone, whereas partial-thickness lesions were confined to the articular cartilage. If multiple lesions were present within the same anatomical region, the region was classified according to the deepest lesion. This also applied to regions containing both artificially created and naturally occurring lesions. The final reference classifications were established by C.J.L. and A.H., who were unaware of the observers’ imaging assessments at that time. The gross pathological findings were compiled into a lesion map, which served as a common reference standard for all imaging assessments across observers, imaging modalities and loading conditions.

### 2.5. Image Analysis

Imaging studies were assessed independently by two board-certified veterinary radiologists (GM-D and DB, both Diplomates of the European College of Veterinary Diagnostic Imaging) and an equine clinician (JMR, Doctor of Veterinary Medicine) with 10 years of clinical experience focused on sports medicine and diagnostic imaging, including previous clinical use of DT. Before assessment, all observers completed a standardised familiarisation session using seven DT images that were not included in the study.

All DT and DTA stacks were prepared by A.H. after image reconstruction and before dataset randomisation and blinding. Markedly blurred slices at the beginning and end of the DT and DTA stacks were removed before evaluation. The number excluded depended on the extent of peripheral blurring and was not recorded. All observers received the same prepared stacks and could review every retained slice.

The anonymised DICOM datasets were evaluated using Horos to assess artificially created and naturally occurring cartilage lesions within the predefined anatomical regions shown in Figure 3. Zooming, scrolling and adjustment of window width and level were unrestricted. Multiplanar reconstruction was also permitted for CT and CTA datasets. An online random-number generator was used to assign each dataset a position in a mixed reading sequence across all four imaging protocols and both loading conditions. The observers followed this sequence without separate modality-specific reading sessions or a predefined washout interval. Each imaging dataset was assessed once by each observer. No separate repeat reading session was performed. They were blinded to the number, location and presence of cartilage lesions and to the findings of the gross pathological assessment.

**Figure 4:**
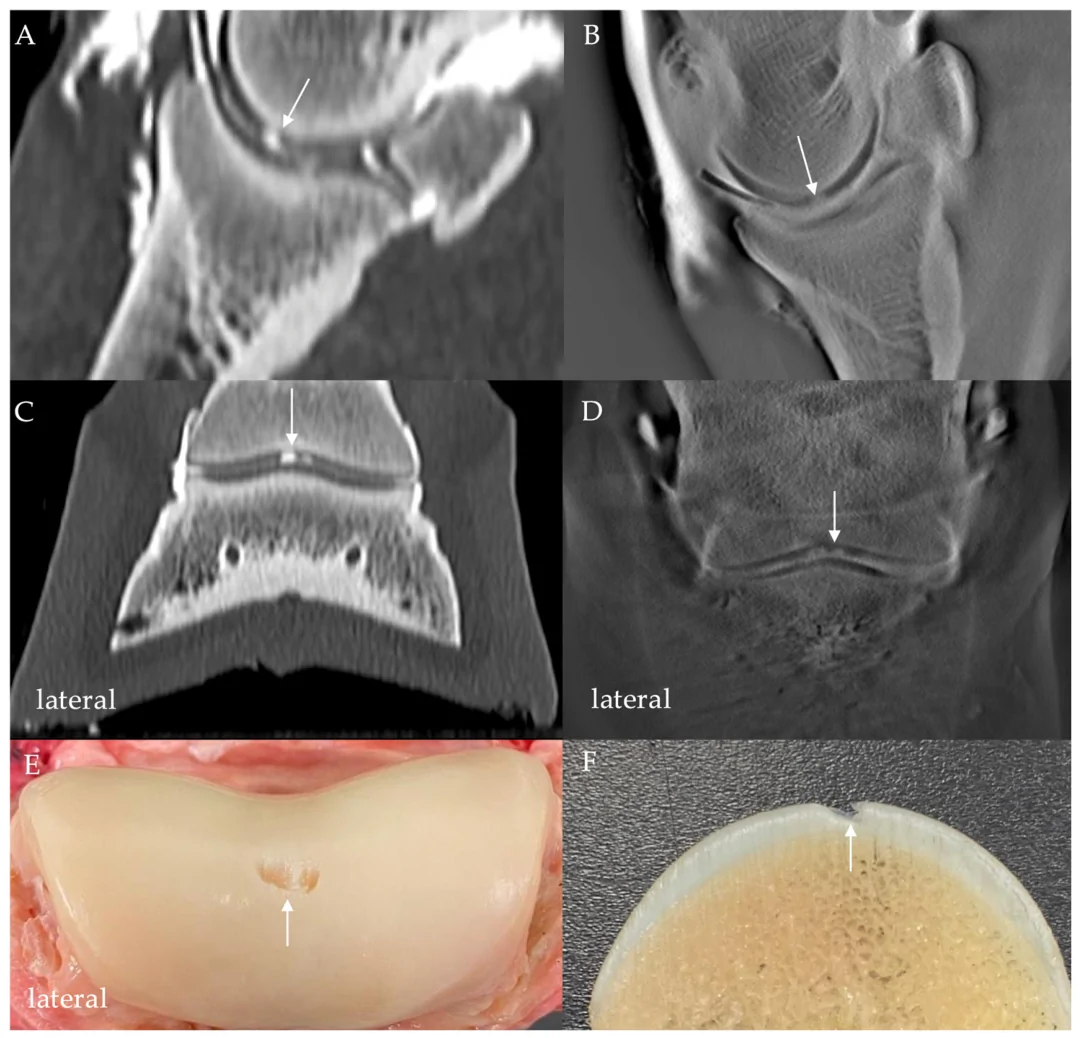
Gross and imaging appearance of an artificially created full-thickness defect on the axial articular surface of MP in the DIP joint. The defect is indicated by an arrow in each image and is visualised by focal filling with contrast medium on the computed tomography arthrography (CTA) and digital tomosynthesis arthrography (DTA) images. (**A**) CTA image under SWB conditions in the sagittal plane. (**B**) DTA image under NWB conditions (LM projection). (**C**) CTA image under SWB conditions in the dorsal plane. (**D**) DTA image under NWB conditions (dorsoproximal-palmarodistal oblique projection). (**E**) Gross evaluation of the articular surface. (**F**) Sagittal section through the defect. Extravasation of contrast medium into the periarticular soft tissues is visible. MP, middle phalanx; DIP, distal interphalangeal; SWB, simulated weight-bearing; NWB, non-weight-bearing; LM, lateromedial.

**Figure 5:**
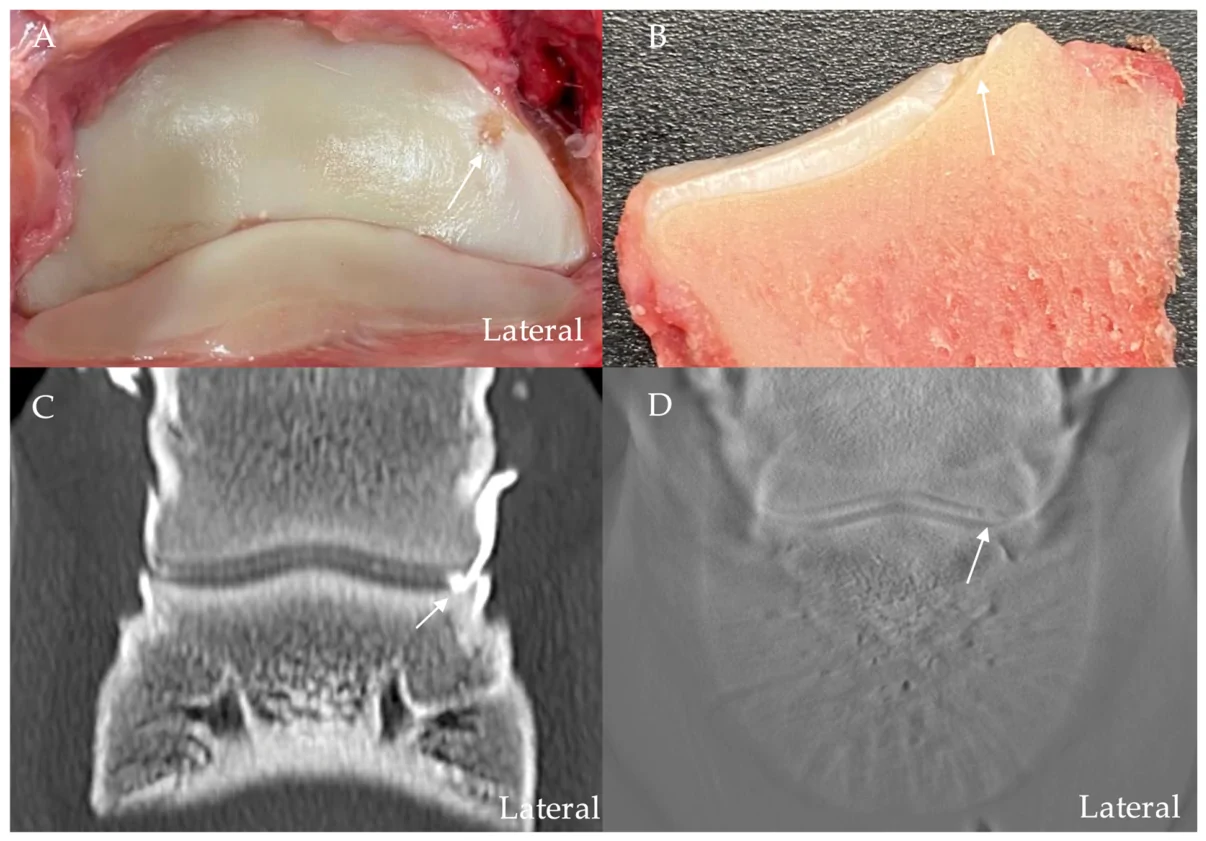
Gross and imaging appearance of an artificially created full-thickness defect on the dorsolateral articular surface of DP. The defect is indicated by an arrow in each image. (**A**) Gross image of the articular surface. (**B**) Parasagittal section through the defect. (**C**) CTA image under SWB conditions in the dorsal plane. (**D**) DTA image under NWB conditions using a dorsoproximal-palmarodistal oblique projection. On both CTA and DTA images, the contrast medium within the DIP joint fills the focal depression in the articular surface corresponding to the artificially created defect. DP, distal phalanx; CTA, computed tomography arthrography; DTA, digital tomosynthesis arthrography; SWB, simulated weight-bearing; NWB, non-weight-bearing; DIP, distal interphalangeal.

**Figure 6:**
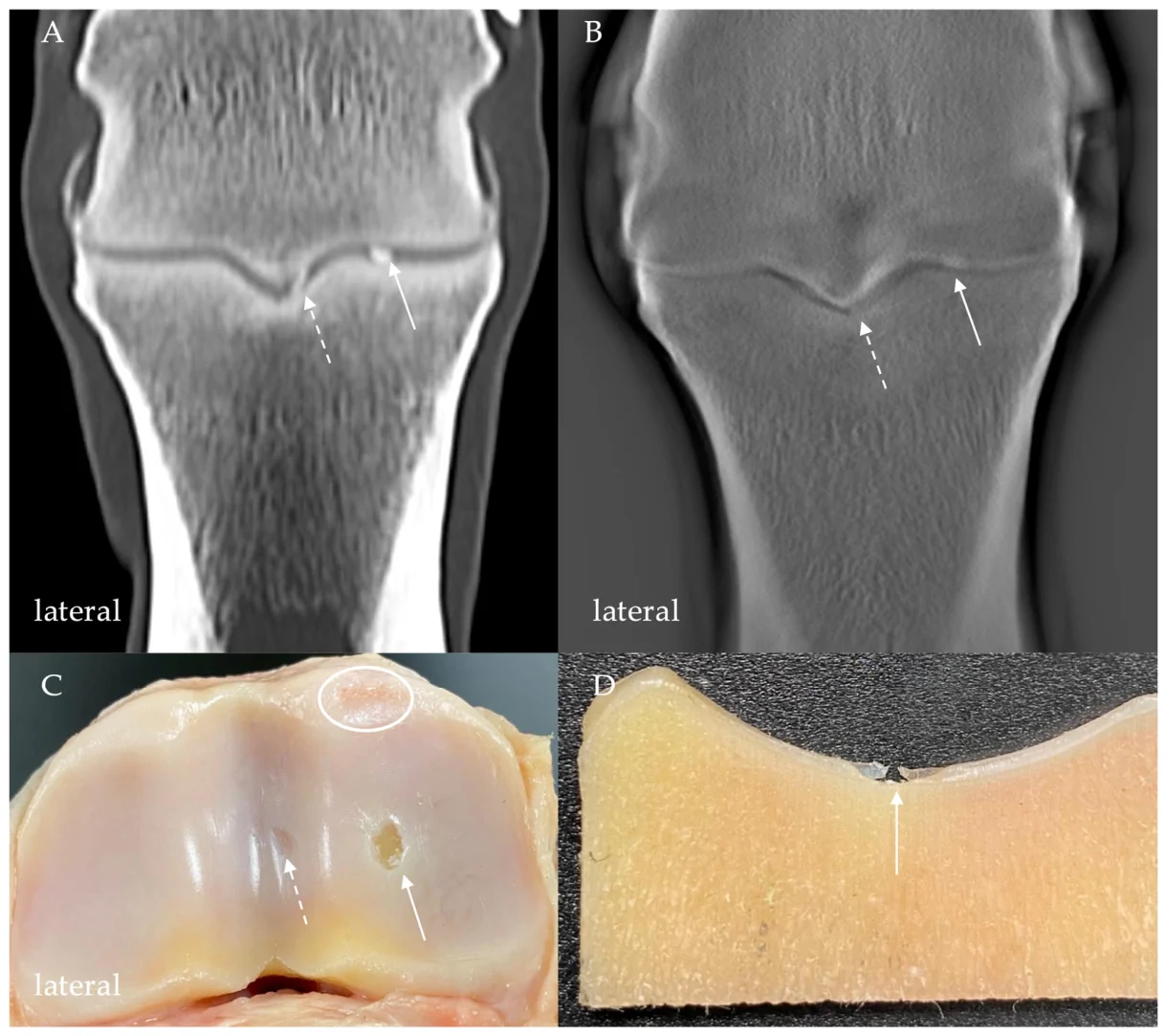
Gross and imaging appearance of artificially created and naturally occurring defects on the articular surface of PP in the MCP joint. The arrow indicates the artificially created full-thickness defect in all four images. The defect is visualised by focal filling with contrast medium on the CTA and DTA images. (**A**) CTA image under SWB conditions in the dorsal plane. (**B**) DTA image under NWB conditions (DPa flexed projection). (**C**) Gross evaluation of the articular surface. (**D**) Parasagittal section through the defect. The dashed arrow denotes an additional, artificially created, partial-thickness defect within the sagittal groove of PP (**A**–**C**). The oval in image (**C**) outlines a naturally occurring full-thickness cartilage lesion. PP, proximal phalanx; MCP, metacarpophalangeal; CTA, computed tomography arthrography; DTA, digital tomosynthesis arthrography; SWB, simulated weight-bearing; NWB, non-weight-bearing; DPa, dorsopalmar.

**Figure 7:**
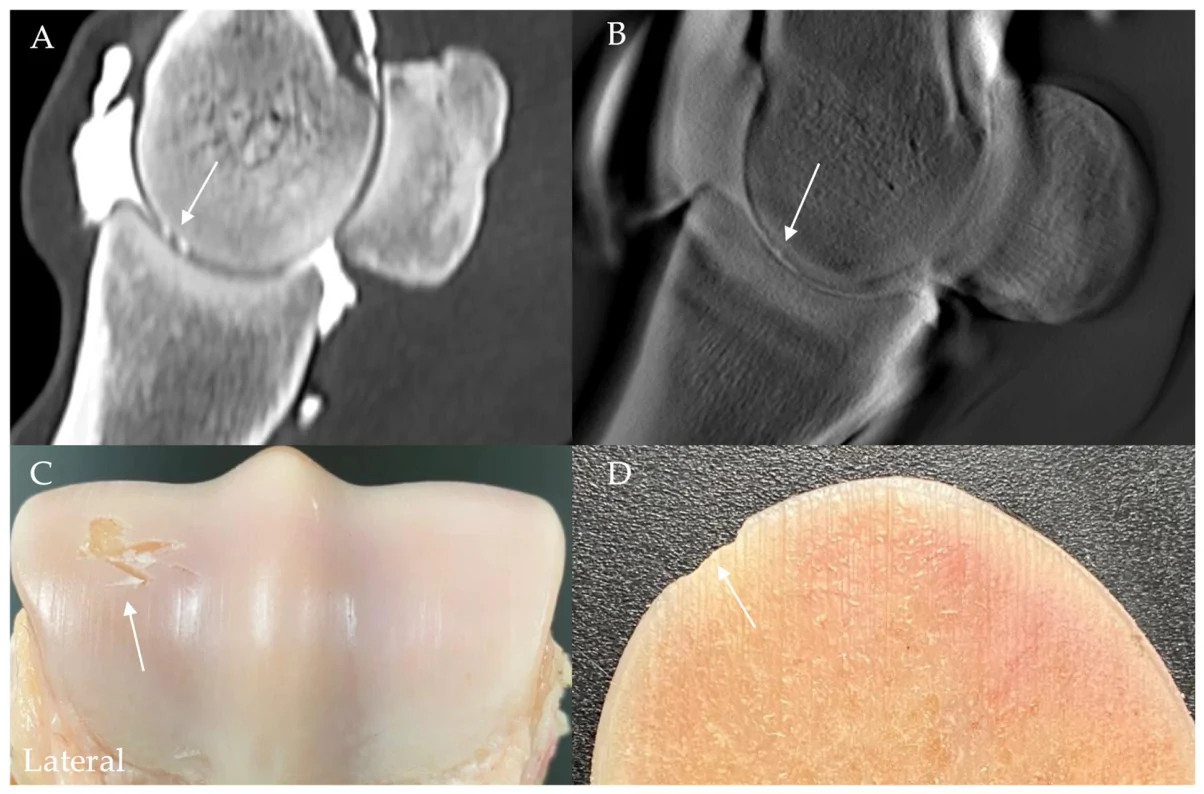
Gross and imaging appearance of an artificially created full-thickness defect on the dorsolateral articular surface of MC3 in the MCP joint. The defect is indicated by an arrow in each image and is visualised by focal filling with contrast medium on CTA and DTA images. (**A**) CTA image under SWB conditions in the sagittal plane. (**B**) DTA image under SWB conditions (LM projection). (**C**) Gross image of the articular surface. (**D**) Parasagittal section through the defect. Extravasation of contrast medium into the periarticular soft tissues is visible. MC3, third metacarpal bone; MCP, metacarpophalangeal; CTA, computed tomography arthrography; DTA, digital tomosynthesis arthrography; SWB, simulated weight-bearing; LM, lateromedial.

Each anatomical region was assigned to one of four diagnostic categories by the observers: ‘no lesion’ = 0, ‘possible lesion’ = 1, ‘partial-thickness lesion’ = 2, or ‘full-thickness lesion’ = 3. A full-thickness lesion was defined as a cartilage defect extending to the subchondral bone, whereas a partial-thickness lesion remained confined to the articular cartilage. A ‘possible lesion’ was recorded when a subtle focal irregularity of the articular cartilage surface suggested a cartilage defect. Representative examples of the gross pathological appearance of the lesions and the corresponding CTA and DTA findings are shown in Figure 4, Figure 5, Figure 6 and Figure 7. Additional examples are provided in the Supplementary Materials (Figures S1–S4).

### 2.6. Statistical Analysis

The evaluations of the three observers were compiled into a consolidated Microsoft Excel spreadsheet (Microsoft Corporation, Redmond, WA, USA) following decoding of the anonymised data. Statistical analyses were performed using IBM SPSS Statistics version 29.0 (IBM Corp., Armonk, NY, USA) and Stata version 19.5 (StataCorp LLC, College Station, TX, USA).

Diagnostic performance was evaluated at the level of the anatomical region. Each imaging assessment represented the classification of one region by one observer for a given imaging modality and loading condition. Each assessment was compared with the gross pathological classification of the corresponding region. For binary analysis, an imaging assessment was classified as positive if the observer recorded ‘possible lesion’, ‘partial-thickness lesion’ or ‘full-thickness lesion’. The ‘possible lesion’ category was included in the positive group because it represented an imaging finding suggestive of cartilage damage. Assessments recorded as ‘no lesion’ were classified as negative. This threshold therefore included suspected as well as definite cartilage lesions.

Sensitivity and specificity were calculated separately for each observer, using gross examination as the reference. Mean sensitivity and specificity were calculated as the arithmetic means of the three observer-specific estimates. Interobserver agreement among the three observers was assessed using Fleiss’ κ and Gwet’s first-order agreement coefficient (AC1) for the binary classifications of lesion absence or presence. Calculations were performed separately for each imaging modality and loading condition across all 384 anatomical regions of the MCP and DIP joints. Agreement with gross examination was assessed separately using Cohen’s κ, pooling the three observers’ assessments within each imaging modality and loading condition. Complete agreement corresponds to a value of 1 for all three coefficients. For Cohen’s κ and Fleiss’ κ, 0 indicates agreement expected by chance, and negative values indicate less agreement than expected by chance. The chance correction for Gwet’s AC1 differs from that for κ. All coefficients were interpreted with their 95% confidence intervals (CI). For sensitivity, specificity and all agreement coefficients, 95% confidence intervals were calculated using a horse-level cluster bootstrap with 10,000 replications and the percentile method. Horses were resampled with replacement, with all observations from each selected horse retained together.

Two mixed-effects logistic regression models were fitted in Stata (version 19.5) to evaluate the odds of true-positive classification (model 1) and true-negative classification (model 2). Both models included imaging modality, loading condition, contrast medium administration and observer as fixed effects. Imaging modality was classified as CT-based (CT and CTA) or DT-based (DT and DTA), with contrast medium administration included as a separate factor. Random intercepts were specified for horses, limbs nested within horses, and observer–limb combinations. Model 1 included only imaging assessments of regions classified as positive according to the reference standard, whereas model 2 included only assessments of regions classified as negative. Correct classifications were coded as 1 and incorrect classifications as 0. Results are presented as adjusted odds ratios (ORs) with 95% confidence intervals and *p* values. Each OR was adjusted for the other fixed effects included in the model. *p* values < 0.05 were considered statistically significant.

## 3. Results

Gross examination identified cartilage lesions in 141 of the 384 anatomical regions. The remaining 243 regions had no cartilage lesions. Of the lesions used to determine the classifications of the 141 positive regions, 89 were artificially created and 52 were naturally occurring. The numbers of true-positive, true-negative, false-positive and false-negative assessments for each imaging modality, loading condition and observer are provided in the Supplementary Materials (Table S2).

Observer-specific and mean sensitivities and specificities, with 95% confidence intervals, are summarised in Table 1. Mean sensitivity was highest for CTA under SWB conditions (60.8%), followed by CTA under NWB conditions (57.0%). Mean sensitivities for DTA were 29.1% under NWB conditions and 14.7% under SWB conditions. Non-contrast CT had lower mean sensitivities (CT NWB, 15.4%; CT SWB, 13.0%), whereas non-contrast DT had the lowest mean sensitivities (DT NWB, 1.7%; DT SWB, 0.5%). Across all three observers, CTA had the highest sensitivity under both SWB and NWB conditions. Observer-specific sensitivities for CTA were 62.4%, 48.2% and 60.3% under NWB conditions and 68.1%, 47.5% and 66.7% under SWB conditions for observers 1, 2 and 3, respectively. None of the imaging modalities had 100% sensitivity for any observer.

Mean specificity was highest for non-contrast DT (NWB, 99.7%; SWB, 99.6%), followed by non-contrast CT (NWB, 99.0%; SWB, 99.2%) and DTA (NWB, 98.1%; SWB, 98.4%). The lowest mean specificities were observed for CTA (NWB, 97.3%; SWB, 96.8%). Observer-specific specificities ranged from 93.4% to 100.0%.

Agreement with gross examination, expressed as pooled Cohen’s κ, and interobserver agreement, expressed as Fleiss’ κ and Gwet’s AC1, are summarised with their 95% confidence intervals in Table 2. Fleiss’ κ values for CT were 0.4667 under NWB conditions and 0.5499 under SWB conditions. The highest point estimates for Fleiss’ κ were observed for CTA (NWB, 0.6136; SWB, 0.6037). Values for DTA were 0.3621 under NWB conditions and 0.3790 under SWB conditions. For non-contrast DT, Fleiss’ κ values were close to zero and slightly negative (NWB, −0.0079; SWB, −0.0044). Gwet’s AC1 point estimates were highest for non-contrast DT (NWB, 0.9841; SWB, 0.9912) and lowest for CTA (NWB, 0.7915; SWB, 0.7693).

For the true-positive model, CT-based imaging was associated with higher odds of correctly identifying a lesion than DT-based imaging (adjusted OR = 6.41, 95% CI 5.23–7.87; *p* < 0.001). Images acquired under SWB conditions were associated with lower odds of a true-positive result than images acquired under NWB conditions (adjusted OR = 0.76, 95% CI 0.63–0.92; *p* = 0.004). Intra-articular contrast medium administration was associated with higher odds of a true-positive result (adjusted OR = 11.41, 95% CI 9.12–14.27; *p* < 0.001). Compared with observer 2, observers 1 and 3 had higher odds of recording a true-positive result (adjusted OR = 2.41, 95% CI 1.90–3.07, and adjusted OR = 2.44, 95% CI 1.92–3.11, respectively; both *p* < 0.001).

For the true-negative model, CT-based imaging was associated with lower odds of correctly identifying a negative region than DT-based imaging (adjusted OR = 0.54, 95% CI 0.35–0.85; *p* = 0.007). No statistically significant difference was observed between SWB and NWB imaging (adjusted OR = 0.98, 95% CI 0.64–1.50; *p* = 0.913). Intra-articular contrast medium administration was associated with lower odds of a true-negative result (adjusted OR = 0.25, 95% CI 0.15–0.43; *p* < 0.001). Compared with observer 2, observer 1 had lower odds of recording a true-negative result (adjusted OR = 0.32, 95% CI 0.17–0.58; *p* < 0.001). The difference between observers 3 and 2 was not statistically significant (adjusted OR = 0.57, 95% CI 0.29–1.10; *p* = 0.093).

The results of the multivariable mixed-effects logistic regression analyses are summarised in Table 3.

## 4. Discussion

To our knowledge, this is the first study to compare DT and DTA with multidetector CT and CTA for the detection of articular cartilage lesions in the equine DIP and MCP joints under both SWB and NWB conditions. The principal findings were that CTA consistently had the highest sensitivity, intra-articular contrast medium administration was associated with higher odds of true-positive classification, and SWB was associated with lower odds of true-positive classification in the main-effects model. The mean sensitivity estimates followed the order CTA, DTA, CT and DT, consistent with our first hypothesis. Suarez Sanchez-Andrade et al. [20] reported a mean CTA sensitivity of 69.9% for experimentally created cartilage defects in the equine carpal joints. In the present study, which included artificial and naturally occurring lesions in the DIP and MCP joints, mean CTA sensitivity reached 60.8% under SWB conditions. In a study of the equine fetlock joint, Hontoir et al. [19] showed that CTA detected articular cartilage defects with a sensitivity of 82%.

Native CT had considerably lower sensitivity, closely matching the value reported by Suarez Sanchez-Andrade et al. [20], who found a sensitivity of 18.1% for the equine carpus. These findings illustrate the limited ability of non-contrast CT to detect cartilage lesions, which is expected given the poor intrinsic contrast between hyaline cartilage and synovial fluid [20].

The higher sensitivity of arthrographic imaging supports the part of our second hypothesis concerning contrast medium administration. In the mixed-effects analysis, intra-articular contrast medium administration was associated with higher odds of true-positive classification (adjusted OR = 11.41, 95% CI 9.12–14.27; *p* < 0.001). Numerically higher sensitivities after intra-articular contrast medium administration have also been reported for acetabular cartilage defects in humans [35] and experimentally created cartilage defects in equine carpal joints [20]. McQuillan et al. [34] also reported improved detection of experimentally created cartilage defects in equine carpal joints with CTA compared with non-contrast CT. Collectively, these findings emphasise that adequate delineation of the cartilage surface by intra-articular contrast medium is important for detecting articular cartilage lesions.

The results did not support a consistent benefit of SWB for lesion detection. Mean CTA sensitivity was numerically higher under SWB than NWB conditions (60.8% versus 57.0%), whereas mean DTA sensitivity was lower (14.7% versus 29.1%). In the main-effects model, SWB was associated with lower odds of true-positive classification (adjusted OR = 0.76, 95% CI 0.63–0.92; *p* = 0.004). This estimate describes the adjusted association across imaging protocols. Several aspects of the experimental protocol may have influenced these findings. First, although the applied load was standardised and based on previously published ex vivo loading protocols [32], it represented only a simplified approximation of physiological weight-bearing. Moreover, intra-articular fluid administration may separate adjacent articular surfaces, allowing contrast medium to delineate the cartilage surface and fill focal defects [20]. During physiological loading, fluid may also become trapped within cartilage surface irregularities [36]. Second, the experimental loading device could not reproduce the dynamic loading associated with locomotion [37], muscle and tendon forces, normal joint motion, or the motion-related changes in intra-articular pressure described in cadaveric equine metatarsophalangeal joints [38]. These factors are likely to influence cartilage deformation and the distribution of contrast medium within the joint. Whether physiological weight-bearing would improve CTA lesion detection could not be determined from this study.

Evrard et al. [39] reported thinner cartilage in the DIP joint, particularly that of the distal phalanx, on weight-bearing MRI of live horses than on non-weight-bearing postmortem MRI. The authors suggested that cartilage compression could make lesions more difficult to detect, although diagnostic sensitivity was not compared. A similar mechanism could have contributed to the lower DTA sensitivity under SWB in the present study. However, cartilage thickness was not measured, and the effects of loading could not be separated from those of joint positioning and projection.

Adequate distribution of the intra-articular contrast medium is likely to be another important determinant of lesion detection. Repeated flexing and extending of the joint after intra-articular contrast medium administration is thought to facilitate the homogeneous distribution of the contrast medium throughout the joint and promote its entry into cartilage defects. Accordingly, the MCP and DIP joints were flexed and extended 20 times following contrast medium injection before CTA acquisition, similar to joint-mobilisation protocols used in previous studies [34,40].

In contrast, native DT had very low sensitivity, with mean values below 2% under both loading conditions. Observers 1 and 2 classified all 384 regions as negative under both loading conditions, resulting in 100% specificity but 0% sensitivity. In a previous cadaveric study, MRI provided better visualisation of articular cartilage than DT [29]. This limitation is not unexpected, as articular cartilage provides only minimal inherent X-ray contrast and is difficult to distinguish from adjacent synovial fluid without intra-articular contrast enhancement [20].

In human medicine, DT has been reported to have greater diagnostic value than conventional radiography for detecting osseous changes associated with OA, including osteophytes and subchondral cysts [23], whereas the present study assessed articular cartilage defects. These findings concern different lesion types and are therefore not directly comparable.

Compared with native DT, DTA had higher mean sensitivity, with values of 29.1% under NWB conditions and 14.7% under SWB conditions. These values remained lower than those obtained with CTA.

To our knowledge, this is the first study to evaluate the diagnostic performance of DTA for detecting articular cartilage lesions in equine joints. In human medicine, however, a technical case report demonstrated that contrast-enhanced tomosynthesis of the hip clearly depicted both a labral tear and adjacent acetabular cartilage loss following intra-articular administration of iodinated contrast medium [41].

Joint positioning differed between the SWB and NWB protocols for both joints, and the projection also differed for the DIP joint. The lower DTA sensitivity observed under SWB conditions therefore cannot be attributed solely to loading or to limitations of the loading apparatus. Future optimisation of acquisition protocols, contrast medium administration, and image reconstruction algorithms may further improve diagnostic performance. The use of DTA could also be evaluated for cartilage assessment in standing horses. However, the possibility of standing acquisition is not unique to DTA, as CT examinations of the distal limb have already been performed in standing horses using a multi-slice helical scanner [22].

Agreement with gross examination was highest for CTA, with pooled Cohen’s κ values of 0.5897 under NWB and 0.6200 under SWB conditions. For non-contrast DT, the corresponding values were close to zero, at 0.0174 and 0.0008, respectively.

The highest point estimates for Fleiss’ κ were observed for CTA (0.6136 under NWB and 0.6037 under SWB conditions), although the corresponding 95% confidence intervals overlapped with those of non-contrast CT. For DTA, Fleiss’ κ values were 0.3621 and 0.3790 under NWB and SWB conditions, respectively. For non-contrast DT, Fleiss’ κ was slightly negative (NWB, −0.0079; SWB, −0.0044), whereas Gwet’s AC1 was highest for this modality (NWB, 0.9841; SWB, 0.9912). Most DT assessments were negative, and the two coefficients calculate the agreement expected by chance differently. The high AC1 values therefore occurred alongside agreement predominantly on negative assessments and very low sensitivity. The ranking of the modalities differed between the two interobserver agreement coefficients.

Mean specificity remained high across all imaging protocols, ranging from 96.8% to 99.7%. The lowest mean specificity was observed for CTA, at 97.3% under NWB and 96.8% under SWB conditions. Intra-articular contrast medium administration was associated with lower odds of correctly classifying regions without cartilage lesions (adjusted OR = 0.25, 95% CI 0.15–0.43; *p* < 0.001). The higher sensitivity of contrast-enhanced imaging was accompanied by more false-positive classifications. Previous equine CTA studies have reported false-positive findings [19,34]. Although cartilage lesions were created underwater to minimise air entry into the joints, intra-articular gas was still present. Intra-articular gas may complicate assessment of cartilage contours and has been discussed as a potential source of false-positive CTA findings [34,40]. Whether it contributed to the false-positive classifications in the present study remains uncertain.

Despite its strengths, this study has several limitations that should be considered. Although 141 cartilage lesion-positive anatomical regions were evaluated, these originated from only 16 limbs from nine horses, limiting the precision and generalisability of the estimates. The horse-level cluster bootstrap accounted for within-horse clustering, but the estimates were still based on only nine horse clusters.

Artificial cartilage lesions were created without direct visualisation of the articular surfaces. Their location, size and depth could not be precisely standardised. Arthroscopic guidance could have improved control in accessible regions. Vanderperren et al. [42] showed that combined arthroscopic approaches with joint flexion and extension allowed visualisation of most MC3 condylar cartilage. Nanoarthroscopy is another potential option. Phillips et al. [43] reported nanoarthroscopic removal of dorsal osteochondral fragments from the DIP joint. Arthroscopic guidance was not used because of concerns about increased contrast medium leakage through additional portals. This concern was not evaluated experimentally, and the study cannot establish whether arthroscopic guidance would have increased contrast medium leakage.

The final reference classification was established by gross examination and sectioning. Variation in lesion morphology and distribution may have influenced detectability and limits generalisability to naturally occurring cartilage disease.

The comparison with gross examination was restricted to lesion presence or absence within predefined anatomical regions and did not assess agreement in lesion size or exact morphological characteristics. ‘Possible lesion’ assessments were classified as positive, so the reported performance includes suspected as well as definite lesions. A true-positive classification therefore did not necessarily indicate correct identification of lesion depth.

The surgical approach used for lesion creation may have affected subsequent arthrographic examinations. Periarticular contrast medium extravasation was documented (Figure 4 and Figure 7) and may have altered joint distension and contrast medium distribution. Its effect on lesion detection was not quantified.

The cadaveric model and static loading protocol did not fully reproduce conditions in a living joint. Active muscle forces were absent, and the same calculated axial load was applied to all limbs without independent force measurement. Although passive motion produces intra-articular pressure changes in cadaveric joints, the absence of blood circulation limits physiological fluid exchange [38]. Joint capsule distensibility and the volume of fluid required to separate opposing articular surfaces may also be affected by the use of cadaveric limbs [44]. Whether these differences from living joints affected contrast medium distribution or lesion detection could not be determined.

For DT and DTA, the comparison between SWB and NWB conditions did not isolate the effect of loading. The NWB acquisitions were obtained with the joints slightly flexed, and the DIP projection differed between conditions. The separate contributions of loading, joint positioning and projection to diagnostic performance could therefore not be determined. Consequently, the regression estimate for SWB should not be interpreted as an isolated effect of loading.

Imaging followed a fixed sequence of native DT, native CT, CTA and DTA, with SWB acquisitions preceding NWB acquisitions for each protocol. Following CTA, DTA was performed without additional contrast medium administration. Possible effects of acquisition order and changes in contrast medium distribution over time could therefore not be separated from differences between protocols.

Despite the randomised reading sequence, recall of findings from other imaging protocols or loading conditions of the same limb cannot be excluded.

Compared with observer 2, observers 1 and 3 had higher adjusted odds of true-positive classification, whereas observer 1 also had lower adjusted odds of true-negative classification. The observers differed in professional background and imaging experience, but the contribution of these differences to reader variability was not assessed.

Finally, the absence of a direct comparison with conventional radiography or MRI limits the scope of the modality evaluation. The present study cannot establish the additional diagnostic value of DT over radiography or its performance relative to MRI.

## 5. Conclusions

In this ex vivo study, CTA had the highest mean sensitivity and pooled Cohen’s κ for agreement with gross examination across the evaluated regions of the equine DIP and MCP joints under both loading conditions. Intra-articular contrast medium administration was associated with higher odds of true-positive classification and lower odds of true-negative classification. Native DT had very low sensitivity for detecting cartilage lesions. The mean sensitivity of DTA was higher than that of native DT but lower than that of CTA. The results did not demonstrate a consistent benefit of SWB for lesion detection. Future in vivo comparative studies are warranted to further define the clinical roles of CTA and DTA in the diagnosis of equine joint disease.

## Figures and Tables

**Table 1:** Sensitivity and specificity for lesion detection under NWB and SWB conditions for three observers using the following imaging modalities: CT, CTA, DT, and DTA. Values are estimates with 95% percentile bootstrap confidence intervals. NE, no informative bootstrap CI due to constant estimates (0% sensitivity or 100% specificity); CI, confidence interval; CT, computed tomography; CTA, computed tomography arthrography; DT, digital tomosynthesis; DTA, digital tomosynthesis arthrography; NWB, non-weight-bearing; SWB, simulated weight-bearing.

Variable	CT		CTA		DT		DTA	
	NWB Estimate 95% CI	SWB Estimate 95% CI	NWB Estimate 95% CI	SWB Estimate 95% CI	NWB Estimate 95% CI	SWB Estimate 95% CI	NWB Estimate 95% CI	SWB Estimate 95% CI
Sensitivity Observer 1	18.4%	14.9%	62.4%	68.1%	0.0%	0.0%	40.4%	16.3%
	(13.3–24.0)	(9.3–21.3)	(54.6–69.7)	(60.7–75.0)	NE	NE	(32.9–47.6)	(12.1–20.4)
Sensitivity Observer 2	9.9%	9.2%	48.2%	47.5%	0.0%	0.0%	12.1%	6.4%
	(6.5–13.6)	(5.8–13.2)	(42.1–54.6)	(41.8–52.7)	NE	NE	(6.9–17.4)	(3.0–10.0)
Sensitivity Observer 3	17.7%	14.9%	60.3%	66.7%	5.0%	1.4%	34.8%	21.3%
	(13.5–22.5)	(11.0–18.4)	(56.1–64.4)	(63.2–70.6)	(2.6–7.2)	(0.0–3.1)	(28.1–42.1)	(15.5–27.7)
Mean Sensitivity	15.4%	13.0%	57.0%	60.8%	1.7%	0.5%	29.1%	14.7%
	(12.0–18.8)	(9.3–16.7)	(53.4–60.5)	(56.7–64.9)	(0.9–2.4)	(0.0–1.0)	(24.5–33.9)	(10.6–18.7)
Specificity Observer 1	97.9%	98.4%	95.5%	93.4%	100.0%	100.0%	97.1%	98.8%
	(96.3–99.3)	(96.7–100.0)	(93.8–97.2)	(89.8–96.8)	NE	NE	(95.3–98.9)	(97.7–99.7)
Specificity Observer 2	100.0%	100.0%	97.5%	99.6%	100.0%	100.0%	98.4%	98.4%
	NE	NE	(95.8–99.1)	(98.8–100.0)	NE	NE	(96.6–100.0)	(96.6–99.7)
Specificity Observer 3	99.2%	99.2%	98.8%	97.5%	99.2%	98.8%	98.8%	97.9%
	(98.1–100.0)	(98.1–100.0)	(97.0–100.0)	(94.9–100.0)	(97.8–100.0)	(97.4–100.0)	(97.0–100.0)	(96.3–99.5)
Mean Specificity	99.0%	99.2%	97.3%	96.8%	99.7%	99.6%	98.1%	98.4%
	(98.3–99.6)	(98.5–99.8)	(96.2–98.2)	(95.3–98.4)	(99.3–100.0)	(99.1–100.0)	(97.3–99.0)	(97.5–99.2)

**Table 2:** Agreement with gross examination (pooled Cohen’s κ) and interobserver agreement (Fleiss’ κ and Gwet’s AC1). Values are estimates with 95% percentile bootstrap confidence intervals. CI, confidence interval; CT, computed tomography; CTA, computed tomography arthrography; DT, digital tomosynthesis; DTA, digital tomosynthesis arthrography; NWB, non-weight-bearing; SWB, simulated weight-bearing; AC1, Gwet’s first-order agreement coefficient.

Variable	CT		CTA		DT		DTA	
	NWB Estimate 95% CI	SWB Estimate 95% CI	NWB Estimate 95% CI	SWB Estimate 95% CI	NWB Estimate 95% CI	SWB Estimate 95% CI	NWB Estimate 95% CI	SWB Estimate 95% CI
Agreement with gross examination: pooled Cohen’s κ	0.1744	0.1485	0.5897	0.6200	0.0174	0.0008	0.3165	0.1574
	(0.1434–0.2057)	(0.1003–0.1914)	(0.5523–0.6238)	(0.5912–0.6581)	(0.0096–0.0235)	(−0.0092–0.0106)	(0.2608–0.3815)	(0.1123–0.2035)
Interobserver agreement: Fleiss’ κ	0.4667	0.5499	0.6136	0.6037	−0.0079	−0.0044	0.3621	0.3790
	(0.3041–0.6197)	(0.3867–0.6760)	(0.5493–0.6930)	(0.5430–0.6743)	(−0.0132–−0.0033)	(−0.0070–−0.0017)	(0.2488–0.4758)	(0.2867–0.4844)
Gwet’s AC1	0.9292	0.9498	0.7915	0.7693	0.9841	0.9912	0.8309	0.9151
	(0.8981–0.9562)	(0.9366–0.9628)	(0.7371–0.8505)	(0.7119–0.8352)	(0.9732–0.9934)	(0.9859–0.9965)	(0.7921–0.8692)	(0.8808–0.9464)

**Table 3:** Results of multivariable mixed-effects logistic regression analyses evaluating associations of imaging modality, loading condition, contrast medium administration and observer with true-positive and true-negative classification. Imaging modality was coded as CT-based (CT and CTA) or DT-based (DT and DTA), with contrast medium administration included as a separate factor. Adjusted odds ratios (ORs) with 95% confidence intervals (CIs) are presented relative to the specified reference categories. Compared with the reference category, ORs greater than 1 indicate higher odds of correct classification, and ORs less than 1 indicate lower odds. CT, computed tomography; CTA, computed tomography arthrography; DT, digital tomosynthesis; DTA, digital tomosynthesis arthrography; NWB, non-weight-bearing; SWB, simulated weight-bearing. *p* values < 0.05 are shown in bold.

Category (*Reference*)	Adjusted Odds Ratio—True-Positive Classification	95% CI	*p* Value	Adjusted Odds Ratio— True-Negative Classification	95% CI	*p* Value
*DT-based imaging*	*1.00 (reference)*			*1.00 (reference)*		
CT-based imaging	6.41	5.23–7.87	**<0.001**	0.54	0.35–0.85	**0.007**
*NWB*	*1.00 (reference)*			*1.00 (reference)*		
SWB	0.76	0.63–0.92	**0.004**	0.98	0.64–1.50	0.913
*Without contrast medium*	*1.00 (reference)*			*1.00 (reference)*		
With contrast medium	11.41	9.12–14.27	**<0.001**	0.25	0.15–0.43	**<0.001**
*Observer 2*	*1.00 (reference)*			*1.00 (reference)*		
Observer 1	2.41	1.90–3.07	**<0.001**	0.32	0.17–0.58	**<0.001**
Observer 3	2.44	1.92–3.11	**<0.001**	0.57	0.29–1.10	0.093

## Data Availability

Data supporting the findings of this study are available from the corresponding author upon reasonable request.

## Supplementary Materials

The following supporting information can be downloaded at: https://www.mdpi.com/article/10.3390/ani16213398/s1, Table S1: Signalment, limb specimens and clinical history of the study horses; Table S2: True-positive, true-negative, false-positive and false-negative assessments by imaging modality, loading condition and observer; Figures S1–S4: Additional gross, computed tomography arthrography and digital tomosynthesis arthrography images of articular cartilage lesions.

## Abbreviations

The following abbreviations are used in this manuscript:

AC1	Gwet’s first-order agreement coefficient
CI	Confidence interval
CT	Computed tomography
CTA	Computed tomography arthrography
DICOM	Digital Imaging and Communications in Medicine
DIP	Distal interphalangeal
DP	Distal phalanx
DPa	Dorsopalmar
DT	Digital tomosynthesis
DTA	Digital tomosynthesis arthrography
LM	Lateromedial
MC3	Third metacarpal bone
MCP	Metacarpophalangeal
MP	Middle phalanx
MRI	Magnetic resonance imaging
NWB	Non-weight-bearing
OA	Osteoarthritis
OR	Odds ratio
PP	Proximal phalanx
Q1	First quartile
Q3	Third quartile
SWB	Simulated weight-bearing

## References

[1] Mackay-Smith M.P. (1962). Pathogenesis and pathology of equine osteoarthritis. J. Am. Vet. Med. Assoc..

[2] Neundorf R.H., Lowerison M.B., Cruz A.M., Thomason J.J., McEwen B.J., Hurtig M.B. (2010). Determination of the prevalence and severity of metacarpophalangeal joint osteoarthritis in Thoroughbred racehorses via quantitative macroscopic evaluation. Am. J. Vet. Res..

[3] Clegg P., Booth T. (2000). Drugs used to treat osteoarthritis in the horse. In Pract..

[4] Barr E.D., Pinchbeck G.L., Clegg P.D., Boyde A., Riggs C.M. (2009). Post mortem evaluation of palmar osteochondral disease (traumatic osteochondrosis) of the metacarpo/metatarsophalangeal joint in Thoroughbred racehorses. Equine Vet. J..

[5] Cantley C.E.L., Firth E.C., Delahunt J.W., Pfeiffer D.U., Thompson K.G. (1999). Naturally occurring osteoarthritis in the metacarpophalangeal joints of wild horses. Equine Vet. J..

[6] van Zadelhoff C., Schwarz T., Smith S., Engerand A., Taylor S. (2020). Identification of naturally occurring cartilage damage in the equine distal interphalangeal joint using low-field magnetic resonance imaging and magnetic resonance arthrography. Front. Vet. Sci..

[7] Dyson S.J. (1991). Lameness due to pain associated with the distal interphalangeal joint: 45 cases. Equine Vet. J..

[8] Kristiansen K.K., Kold S.E. (2007). Multivariable analysis of factors influencing outcome of 2 treatment protocols in 128 cases of horses responding positively to intra-articular analgesia of the distal interphalangeal joint. Equine Vet. J..

[9] McKnight A.L., Posh J. (2012). Articular cartilage lesions seen with MRI at 0.25T in the distal equine limb. J. Equine Vet. Sci..

[10] Hunt M.D.N. (1965). Traumatic arthritis in young Thoroughbreds. Proc. R. Soc. Med..

[11] McIlwraith C.W. (1982). Current concepts in equine degenerative joint disease. J. Am. Vet. Med. Assoc..

[12] McIlwraith C.W., Frisbie D.D., Kawcak C.E. (2012). The horse as a model of naturally occurring osteoarthritis. Bone Jt. Res..

[13] Nelson B.B., Kawcak C.E., Barrett M.F., McIlwraith C.W., Grinstaff M.W., Goodrich L.R. (2018). Recent advances in articular cartilage evaluation using computed tomography and magnetic resonance imaging. Equine Vet. J..

[14] Barton C.K., Nelson B.B., Goodrich L.R. (2026). Review of articular cartilage repair techniques and their application in the horse. Equine Vet. J..

[15] Hurtig M.B., Fretz P.B., Doige C.E., Schnurr D.L. (1988). Effects of lesion size and location on equine articular cartilage repair. Can. J. Vet. Res..

[16] Nickels F.A., Sande R. (1982). Radiographic and arthroscopic findings in the equine stifle. J. Am. Vet. Med. Assoc..

[17] Garrett K.S. (2022). When radiography and ultrasonography are not enough: The use of computed tomography and magnetic resonance imaging for equine lameness cases. J. Am. Vet. Med. Assoc..

[18] Tucker R.L., Sande R.D. (2001). Computed tomography and magnetic resonance imaging in equine musculoskeletal conditions. Vet. Clin. N. Am. Equine Pract..

[19] Hontoir F., Nisolle J.-F., Meurisse H., Simon V., Tallier M., Vanderstricht R., Antoine N., Piret J., Clegg P., Vandeweerd J.-M. (2014). A comparison of 3-T magnetic resonance imaging and computed tomography arthrography to identify structural cartilage defects of the fetlock joint in the horse. Vet. J..

[20] Suarez Sanchez-Andrade J., Richter H., Kuhn K., Bischofberger A.S., Kircher P.R., Hoey S. (2018). Comparison between magnetic resonance imaging, computed tomography, and arthrography to identify artificially induced cartilage defects of the equine carpal joints. Vet. Radiol. Ultrasound.

[21] Moser T., Khoury V., Harris P.G., Bureau N.J., Cardinal E., Dosch J.C. (2009). MDCT arthrography or MR arthrography for imaging the wrist joint?. Semin. Musculoskelet. Radiol..

[22] Mageed M. (2022). Standing computed tomography of the equine limb using a multi-slice helical scanner: Technique and feasibility study. Equine Vet. Educ..

[23] Hayashi D., Xu L., Roemer F.W., Hunter D.J., Li L., Katur A.M., Guermazi A. (2012). Detection of osteophytes and subchondral cysts in the knee with use of tomosynthesis. Radiology.

[24] Ottenin M.-A., Jacquot A., Grospretre O., Noël A., Lecocq S., Louis M., Blum A. (2012). Evaluation of the diagnostic performance of tomosynthesis in fractures of the wrist. AJR Am. J. Roentgenol..

[25] Aoki T., Fujii M., Yamashita Y., Takahashi H., Oki H., Hayashida Y., Saito K., Tanaka Y., Korogi Y. (2014). Tomosynthesis of the wrist and hand in patients with rheumatoid arthritis: Comparison with radiography and MRI. AJR Am. J. Roentgenol..

[26] Canella C., Philippe P., Pansini V., Salleron J., Flipo R.-M., Cotten A. (2011). Use of tomosynthesis for erosion evaluation in rheumatoid arthritic hands and wrists. Radiology.

[27] Blum A., Noël A., Regent D., Villani N., Gillet R., Gondim Teixeira P. (2018). Tomosynthesis in musculoskeletal pathology. Diagn. Interv. Imaging.

[28] Machida H., Yuhara T., Tamura M., Ishikawa T., Tate E.T., Ueno E., Nye K., Sabol J.M. (2016). Whole-body clinical applications of digital tomosynthesis. Radiographics.

[29] Stewart H.L., Kawcak C.E., Inscoe C.R., Puett C., Lee Y.Z., Lu J., Zhou O.Z., Selberg K.T. (2021). Comparative evaluation of tomosynthesis, computed tomography, and magnetic resonance imaging findings for metacarpophalangeal joints from equine cadavers. Am. J. Vet. Res..

[30] ICRP (2022). ICRP Publication 153: Radiological Protection in Veterinary Practice. Ann. ICRP.

[31] Hahn A., Valdés Vázquez M., Manso-Díaz G., Berner D., Ramos J.M., Doherr M.G., Lischer C.J. (2026). Diagnostic Performance of Digital Tomosynthesis for Detecting Osteophytes and Subchondral Bone Changes in the Equine Distal Interphalangeal and Metacarpophalangeal Joints. Animals.

[32] Viitanen M.J., Wilson A.M., McGuigan H.P., Rogers K.D., May S.A. (2003). Effect of foot balance on the intra-articular pressure in the distal interphalangeal joint in vitro. Equine Vet. J..

[33] Manso Díaz G., López San Román J., Weller R. (2018). A Practical Guide to Equine Radiography.

[34] McQuillan S., Kearney C., Hoey S., Connolly S., Rowan C. (2022). A threshold volume of 10 mL is suggested for detecting articular cartilage defects in equine carpal joints using CT arthrography: Ex vivo pilot study. Vet. Radiol. Ultrasound.

[35] Sutter R., Zubler V., Hoffmann A., Mamisch-Saupe N., Dora C., Kalberer F., Zanetti M., Hodler J., Pfirrmann C.W.A. (2014). Hip MRI: How useful is intraarticular contrast material for evaluating surgically proven lesions of the labrum and articular cartilage?. AJR Am. J. Roentgenol..

[36] Walker P.S., Dowson D., Longfield M.D., Wright V. (1968). “Boosted lubrication” in synovial joints by fluid entrapment and enrichment. Ann. Rheum. Dis..

[37] Ateshian G.A. (2009). The role of interstitial fluid pressurization in articular cartilage lubrication. J. Biomech..

[38] da Graca Macoris D., Bertone A. (2001). Intra-articular pressure profiles of the cadaveric equine fetlock joint in motion. Equine Vet. J..

[39] Evrard L., Audigié F., Bertoni L., Jacquet S., Denoix J.-M., Busoni V. (2019). Low field magnetic resonance imaging of the equine distal interphalangeal joint: Comparison between weight-bearing and non-weight-bearing conditions. PLoS ONE.

[40] Bolz N.M., Sánchez-Andrade J.S., Torgerson P.R., Bischofberger A.S. (2023). Diagnostic performance of multi-detector computed tomography arthrography and 3-Tesla magnetic resonance imaging to diagnose experimentally created articular cartilage lesions in equine cadaver stifles. Animals.

[41] Gazaille R.E., Flynn M.J., Page W., Finley S., van Holsbeeck M. (2011). Technical innovation: Digital tomosynthesis of the hip following intra-articular administration of contrast. Skelet. Radiol..

[42] Vanderperren K., Martens A., Haers H., Duchateau L., Saunders J.H. (2009). Arthroscopic visualisation of the third metacarpal and metatarsal condyles in the horse. Equine Vet. J..

[43] Phillips N.A.I., Fortier L.A., Cable C.S., Colbath A.C. (2026). Standing NanoNeedle Arthroscopy of the Distal Interphalangeal Joint for Removal of Osteochondral Fragments of Distal P2 and the Extensor Process in a Horse. Animals.

[44] Porter E.G., Winter M.D., Sheppard B.J., Berry C.R., Hernandez J.A. (2016). Correlation of articular cartilage thickness measurements made with magnetic resonance imaging, magnetic resonance arthrography, and computed tomographic arthrography with gross articular cartilage thickness in the equine metacarpophalangeal joint. Vet. Radiol. Ultrasound.

