# Supplementary material for: Evaluation of Digital Tomosynthesis for Detecting Articular Cartilage Lesions in Equine Distal Interphalangeal and Metacarpophalangeal Joints: Comparison with Computed Tomography Arthrography
Source: Animals (Basel). 2026 Oct 28;16(21):3398. doi: 10.3390/ani16213398 (PMC13761513; doi:10.3390/ani16213398)
Supplement: Supplementary file 1 [file animals-16-03398-s001.zip › Supplementary Material_Animals_Hahn.pdf]

Evaluation of Digital Tomosynthesis for Detecting Articular Cartilage Lesions in Equine Distal Interphalangeal and Metacarpophalangeal Joints: Comparison with Computed Tomography Arthrography

Alisa Hahn[1,*], Miguel Valdés Vázquez[2], Gabriel Manso-Díaz[3], Dagmar Berner[4], Joana Maia Ramos[2], Marcus G. Doherr[5] and Christoph J. Lischer[1]

[1] Equine Clinic, Veterinary Hospital, School of Veterinary Medicine, Freie Universität Berlin, Oertzenweg 19b, 14163 Berlin, Germany; a.hahn@fu-berlin.de; christoph.lischer@fu-berlin.de
[2] Hospital de Referencia La Equina, Apdo. 110, Camino de Martagina Km 1, 29692 Manilva, Málaga, Spain; miguel@laequina.com; joana.126@hotmail.com
[3] Department of Animal Medicine and Surgery, Faculty of Veterinary Medicine, Complutense University of Madrid, Avenida Puerta de Hierro, s/n, 28040 Madrid, Spain; gmanso@ucm.es
[4] Equine Referral Hospital, Department of Clinical Science and Services, Royal Veterinary College, Hawkshead Lane, North Mymms, Hatfield, Hertfordshire AL9 7TA, UK; dberner@rvc.ac.uk
[5] Institute of Veterinary Epidemiology and Biostatistics, School of Veterinary Medicine, Freie Universität Berlin, Königsweg 67, 14163 Berlin, Germany; marcus.doherr@fu-berlin.de
* Correspondence: a.hahn@fu-berlin.de

Supplementary Material

Table S1. Signalment, limb specimens, and recorded clinical history of the nine horses included in the study. LF, left forelimb; RF, right forelimb.

Horse	Case number(s) and forelimb(s)	Age (years)	Sex	Breed	Body weight (kg)	Recorded clinical history
Horse 1	01 (RF)	20	Gelding	Holsteiner	500	Large-colon displacement and gaseous distension; suspected small-intestinal obstruction; euthanasia, surgery not authorised
Horse 2	02 (LF)<br>03 (RF)	15	Mare	Arabian	480	Caecal torsion; intraoperative euthanasia
Horse 3	04 (LF)	19	Gelding	Hanoverian	575	Severe enteritis; gastric rupture; euthanasia because of a poor prognosis
Horse 4	05 (RF)<br>06 (LF)	7	Stallion	German Sport Horse	570	Colon gaseous distension and gastric overload; suspected right-sided displacement; euthanasia, surgery not authorised
Horse 5	07 (RF)<br>08 (LF)	12	Gelding	Hanoverian	500	Small-intestinal obstruction; euthanasia, surgery not authorised
Horse 6	09 (RF)<br>10 (LF)	12	Mare	German Sport Horse	500	Colitis; suspected small-intestinal ileus; euthanasia because of a poor prognosis
Horse 7	11 (RF)<br>12 (LF)	3	Mare	Thoroughbred	480	Severe impaction of the ascending and descending colon; displacement of the ascending colon into the nephrosplenic space; postoperative colitis; euthanasia because of a poor prognosis
Horse 8	13 (RF)<br>14 (LF)	13	Gelding	Pura Raza Española	500	Suspected intoxication; haemolytic anaemia of unknown aetiology; euthanasia because of a poor prognosis
Horse 9	15 (RF)<br>16 (LF)	17	Mare	Thoroughbred	650	Large-colon gaseous distension, small-intestinal dilatation; horse was unable to rise due to severe neurological deficits; euthanasia because of a poor prognosis

Table S2. Numbers of true-positive (TP), true-negative (TN), false-positive (FP) and false-negative (FN) cartilage lesion assessments for each imaging modality, loading condition and observer, using gross examination as the reference standard. Imaging scores 1–3 were classified as positive. CT, computed tomography; CTA, computed tomographic arthrography; DT, digital tomosynthesis; DTA, digital tomosynthesis arthrography; NWB, non-weight-bearing; SWB, simulated weight-bearing; N, total number of assessed regions.

Modality	Loading	Observer	TP	TN	FP	FN	N	Reference positive regions	Reference negative regions
CT	NWB	1	26	238	5	115	384	141	243
CT	NWB	2	14	243	0	127	384	141	243
CT	NWB	3	25	241	2	116	384	141	243
CT	SWB	1	21	239	4	120	384	141	243
CT	SWB	2	13	243	0	128	384	141	243
CT	SWB	3	21	241	2	120	384	141	243
CTA	NWB	1	88	232	11	53	384	141	243
CTA	NWB	2	68	237	6	73	384	141	243
CTA	NWB	3	85	240	3	56	384	141	243
CTA	SWB	1	96	227	16	45	384	141	243
CTA	SWB	2	67	242	1	74	384	141	243
CTA	SWB	3	94	237	6	47	384	141	243
DT	NWB	1	0	243	0	141	384	141	243
DT	NWB	2	0	243	0	141	384	141	243
DT	NWB	3	7	241	2	134	384	141	243
DT	SWB	1	0	243	0	141	384	141	243

DT	SWB	2	0	243	0	141	384	141	243
DT	SWB	3	2	240	3	139	384	141	243
DTA	NWB	1	57	236	7	84	384	141	243
DTA	NWB	2	17	239	4	124	384	141	243
DTA	NWB	3	49	240	3	92	384	141	243
DTA	SWB	1	23	240	3	118	384	141	243
DTA	SWB	2	9	239	4	132	384	141	243
DTA	SWB	3	30	238	5	111	384	141	243
