# Supplementary material for: Evaluation of Digital Tomosynthesis for Detecting Articular Cartilage Lesions in Equine Distal Interphalangeal and Metacarpophalangeal Joints: Comparison with Computed Tomography Arthrography
Source: Animals (Basel). 2026 Oct 28;16(21):3398. doi: 10.3390/ani16213398 (PMC13761513; doi:10.3390/ani16213398)
Supplement: Supplementary file 1 [file animals-16-03398-s001.zip › Supplementary Material_Figures_Animals_Hahn.pdf]

Evaluation of Digital Tomosynthesis for Detecting Articular Cartilage Lesions in Equine Distal Interphalangeal and Metacarpophalangeal Joints: Comparison with Computed Tomography Arthrography

Alisa Hahn[1,*], Miguel Valdés Vázquez[2], Gabriel Manso-Díaz[3], Dagmar Berner[4], Joana Maia Ramos[2], Marcus G. Doherr[5] and Christoph J. Lischer[1]

[1] Equine Clinic, Veterinary Hospital, School of Veterinary Medicine, Freie Universität Berlin, Oertzenweg 19b, 14163 Berlin, Germany; a.hahn@fu-berlin.de; christoph.lischer@fu-berlin.de

[2] Hospital de Referencia La Equina, Apdo. 110, Camino de Martagina Km 1, 29692 Manilva, Málaga, Spain; miguel@laequina.com; joana.126@hotmail.com

[3] Department of Animal Medicine and Surgery, Faculty of Veterinary Medicine, Complutense University of Madrid, Avenida Puerta de Hierro, s/n, 28040 Madrid, Spain; gmanso@ucm.es

[4] Equine Referral Hospital, Department of Clinical Science and Services, Royal Veterinary College, Hawkshead Lane, North Mymms, Hatfield, Hertfordshire AL9 7TA, UK; dberner@rvc.ac.uk

[5] Institute of Veterinary Epidemiology and Biostatistics, School of Veterinary Medicine, Freie Universität Berlin, Königsweg 67, 14163 Berlin, Germany; marcus.doherr@fu-berlin.de

* Correspondence: a.hahn@fu-berlin.de

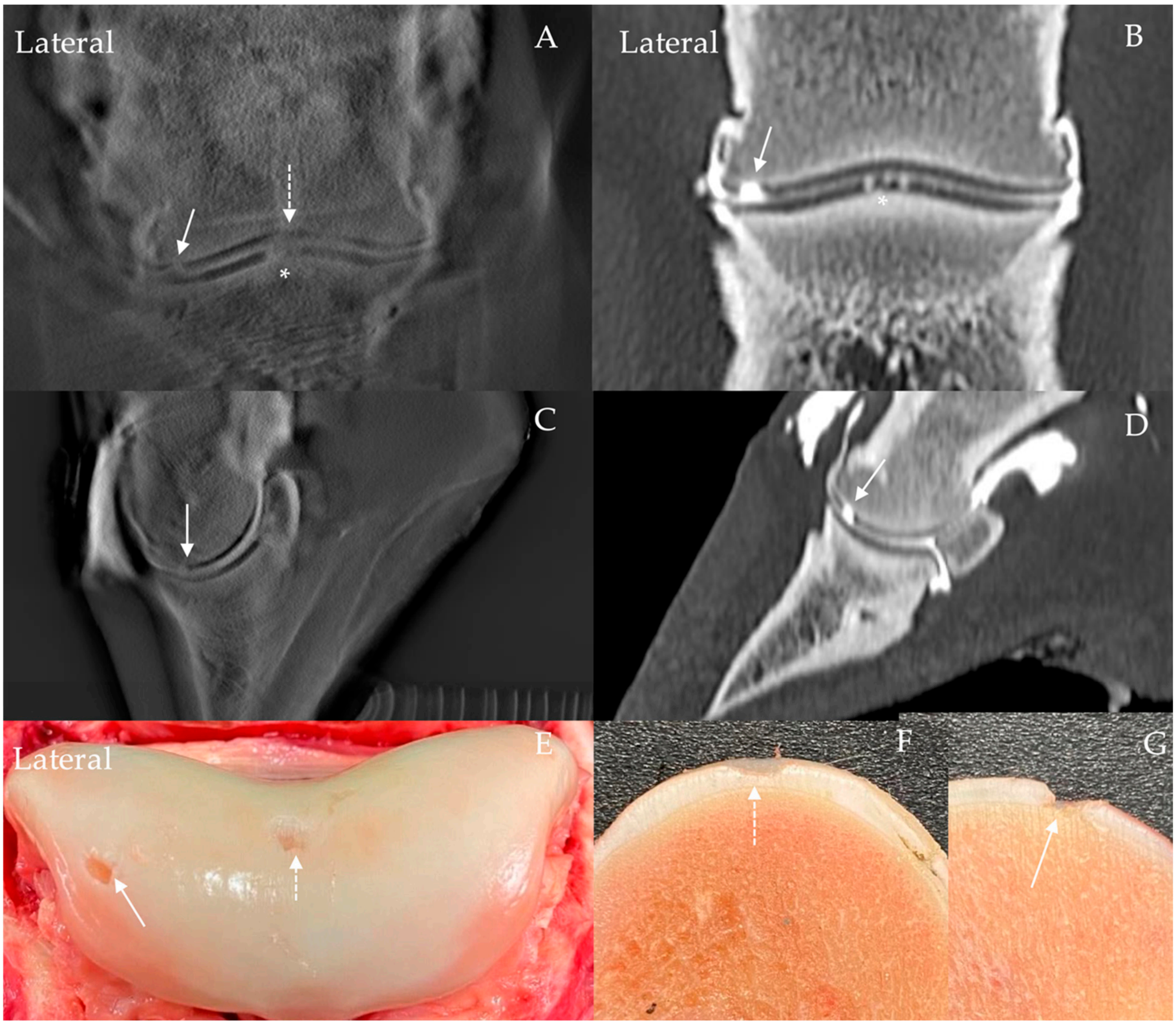

Figure S1. Gross and imaging appearance of artificially created full-thickness and partial-thickness defects on the articular surface of the middle phalanx (MP) in the distal interphalangeal (DIP) joint. The solid arrows indicate the lateral full-thickness defect, and the dashed arrows indicate the axial partial-thickness defect. (A) Digital tomosynthesis arthrography (DTA) image under non-weight-bearing (NWB) conditions (dorsoproximal–palmarodistal oblique projection). (B) Computed tomography arthrography (CTA) image under simulated weight-bearing (SWB) conditions in the dorsal plane. (C) DTA image under NWB conditions (lateromedial projection). (D) CTA image under SWB conditions in the sagittal plane. (E) Gross evaluation of the articular surface. (F) Section through the axial partial-thickness defect. (G) Section through the lateral full-thickness defect. The asterisks in A and B indicate an axial defect of the distal phalanx (DP). The lateral aspect is indicated in A, B and E.

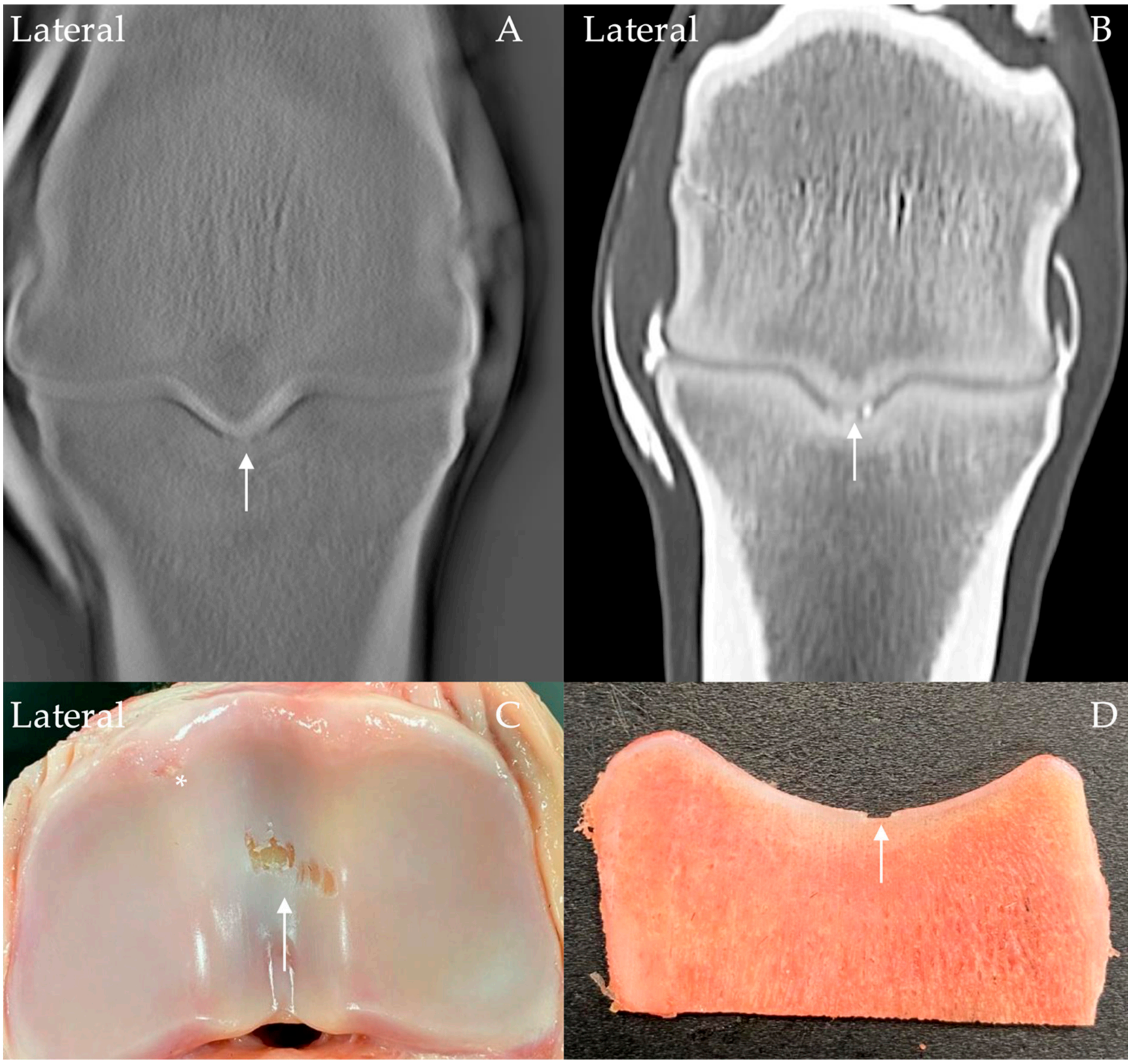

Figure S2. Gross and imaging appearance of artificially created and naturally occurring cartilage defects on the articular surface of the proximal phalanx (PP) in the metacarpophalangeal (MCP) joint. The arrow indicates the artificially created full-thickness defect within the sagittal groove in all four images. (A) Digital tomosynthesis arthrography (DTA) image under non-weight-bearing (NWB) conditions (dorsopalmar projection with the joint slightly flexed). (B) Computed tomography arthrography (CTA) image under simulated weight-bearing (SWB) conditions in the dorsal plane. (C) Gross evaluation of the articular surface. (D) Section through the full-thickness defect. The asterisk in C indicates an additional, naturally occurring partial-thickness cartilage lesion. The lateral aspect is indicated in A–C.

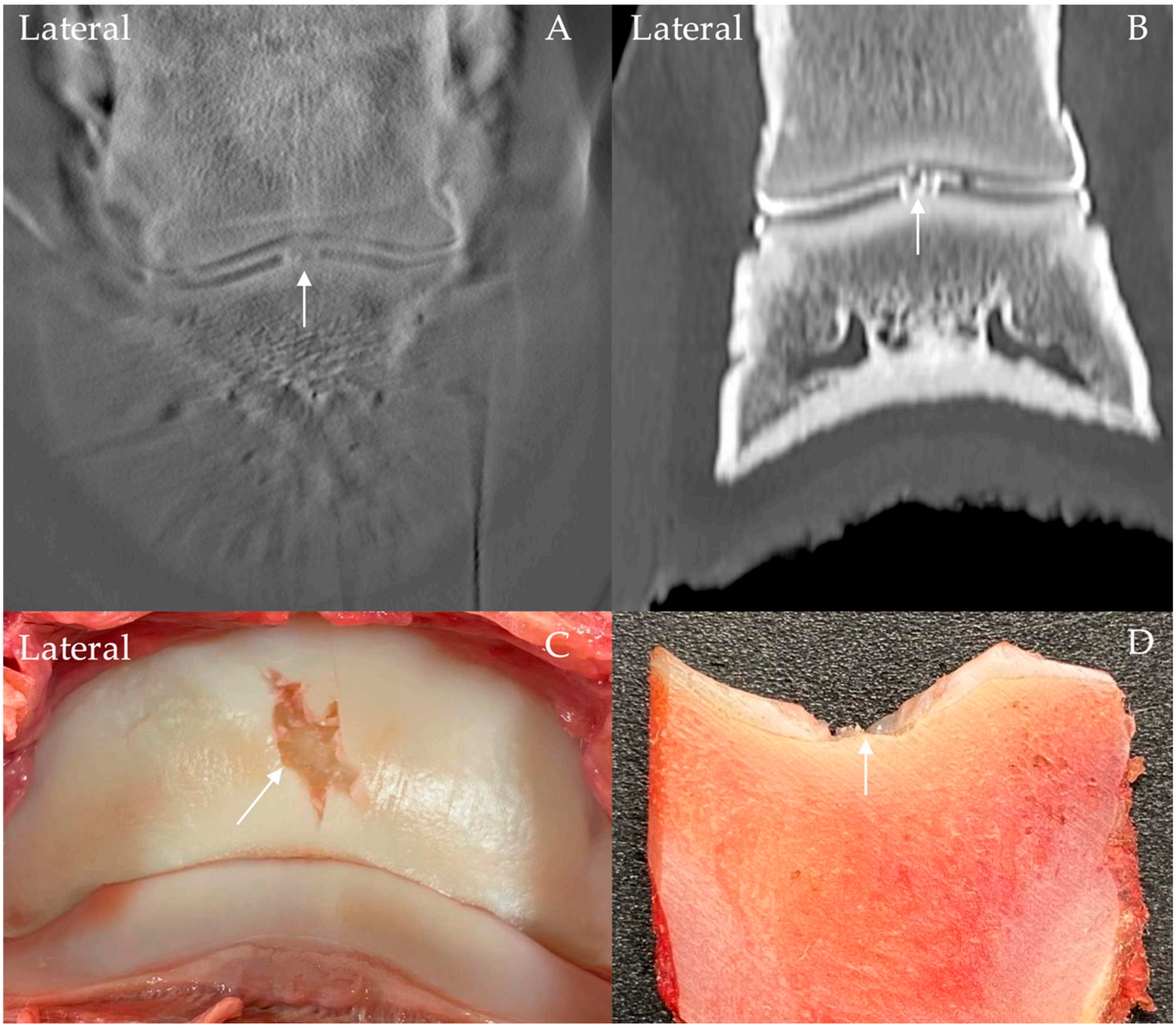

Figure S3. Gross and imaging appearance of an artificially created full-thickness defect on the axial articular surface of the distal phalanx (DP) in the distal interphalangeal (DIP) joint. The defect is indicated by an arrow in each image. (A) Digital tomosynthesis arthrography (DTA) image under non-weight-bearing (NWB) conditions (dorsoproximal–palmarodistal oblique projection). (B) Computed tomography arthrography (CTA) image under simulated weight-bearing (SWB) conditions in the dorsal plane. (C) Gross evaluation of the articular surface. (D) Section through the defect. The lateral aspect is indicated in A–C.

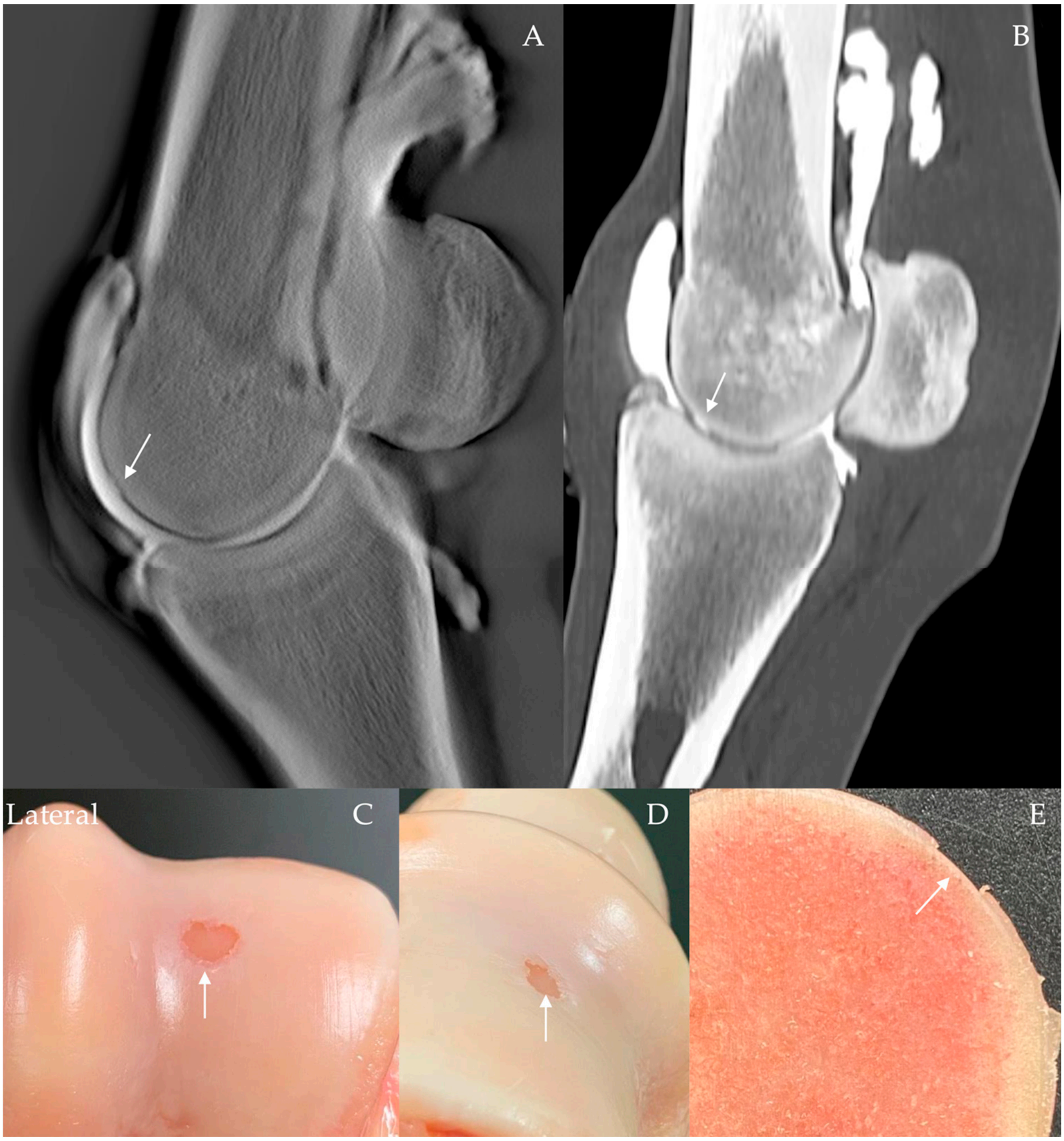

Figure S4. Gross and imaging appearance of a naturally occurring full-thickness cartilage defect on the dorsomedial articular surface of the third metacarpal bone (MC3) in the metacarpophalangeal (MCP) joint. The defect was associated with an osteochondral fragment at the dorsomedial aspect of the proximal phalanx (PP) and is indicated by an arrow in each image. (A) Digital tomosynthesis arthrography (DTA) image under non-weight-bearing (NWB) conditions (lateromedial projection). (B) Computed tomography arthrography (CTA) image under simulated weight-bearing (SWB) conditions in the sagittal plane. (C,D) Gross evaluation of the articular surface from two different views. (E) Section through the defect. The lateral aspect is indicated in C.
